# A systematic review of childhood maltreatment and DNA methylation: candidate gene and epigenome-wide approaches

**DOI:** 10.1038/s41398-021-01207-y

**Published:** 2021-02-19

**Authors:** Stephanie H. Parade, Lindsay Huffhines, Teresa E. Daniels, Laura R. Stroud, Nicole R. Nugent, Audrey R. Tyrka

**Affiliations:** 1 Initiative on Stress, Trauma, and Resilience, Department of Psychiatry and Human Behavior, Warren Alpert Medical School, Brown University, Providence, RI USA; 2 Bradley/Hasbro Children’s Research Center, E. P. Bradley Hospital, East Providence, RI USA; 3 Laboratory for Clinical and Translational Neuroscience, Butler Hospital, Providence, RI USA; 4 Center for Behavioral and Preventive Medicine, The Miriam Hospital, Providence, RI USA

**Keywords:** Human behaviour, Epigenetics and behaviour

## Abstract

Childhood maltreatment is a major risk factor for chronic and severe mental and physical health problems across the lifespan. Increasing evidence supports the hypothesis that maltreatment is associated with epigenetic changes that may subsequently serve as mechanisms of disease. The current review uses a systematic approach to identify and summarize the literature related to childhood maltreatment and alterations in DNA methylation in humans. A total of 100 empirical articles were identified in our systematic review of research published prior to or during March 2020, including studies that focused on candidate genes and studies that leveraged epigenome-wide data in both children and adults. Themes arising from the literature, including consistent and inconsistent patterns of results, are presented. Several directions for future research, including important methodological considerations for future study design, are discussed. Taken together, the literature on childhood maltreatment and DNA methylation underscores the complexity of transactions between the environment and biology across development.

## Introduction

Childhood maltreatment is a highly prevalent public health problem that often has devastating effects on physical and mental health. There are now numerous studies linking childhood maltreatment and other early adversities to nearly all forms of mental illness, as well as chronic medical conditions that cut across multiple organ systems^1–7^. Unfortunately, the negative sequelae of childhood maltreatment often begin early in life, and persist across adulthood^1,6^, posing risk for premature mortality. Indeed, adults with numerous adverse experiences in childhood die nearly 20 years earlier than those with no early adversity^1^. Understanding the mechanisms of biological influence is therefore critical to developing innovative targets for intervention to enhance health outcomes among highly vulnerable children with this major adverse exposure.

Over the past decade a rapidly growing body of literature has underscored the significant role of epigenetics in the sequelae of childhood maltreatment. Epigenetic processes allow the body to respond to environmental influences by altering gene expression through chemical modifications that regulate chromatin structure and/or DNA accessibility without inducing changes to the DNA sequence^8^. DNA methylation is among the most commonly studied epigenetic processes and involves the addition of a methyl group at sites in the DNA where a cytosine nucleotide occurs next to a guanine nucleotide (CpG dinucleotides). Initial work focused on understanding associations of early life stress and DNA methylation examined these processes in rodents and found that low levels of maternal care (licking and grooming and arched-back nursing) was associated with greater methylation of the glucocorticoid receptor (GR) gene in offspring^9,10^. Furthermore, this work suggested that methylation was a mechanism of the effect of maternal care on the offspring HPA stress response. Emerging from this groundbreaking work with animal models, some of the earliest studies in humans documenting altered DNA methylation in association with childhood maltreatment focused on the glucocorticoid receptor (GR) gene, *NR3C1*, which modifies responsiveness of the HPA axis to stress exposure. Our group was the first to demonstrate altered leukocyte DNA methylation of *NR3C1* in adults with childhood maltreatment^11^, associations of *NR3C1* methylation with behavior problems and symptoms in children with early adversity^12^, and maltreatment as a predictor of change in *NR3C1* methylation over time^13^. In 2016, we completed a literature review focused on altered methylation of glucocorticoid signaling genes in association with childhood maltreatment^14^. Several other recent reviews have also focused on methylation of glucocorticoid signaling genes^15–17^, as well as methylation of genes associated with the serotonin system^18,19^ in relation to adverse exposures. We expand upon these prior reviews to consider the recent explosion of research in this area that has examined additional genes as well as epigenome-wide effects. Cecil and colleagues recently conducted a systematic review of research focused on childhood maltreatment, specifically experiences of abuse and neglect, and DNA methylation^20^. The current review provides an important addition to the literature by utilizing a broader conceptualization of childhood maltreatment that includes any experience that involved potential for harm to the child. Our systematic approach to identify and summarize the literature related to childhood maltreatment and other adversities in association with DNA methylation captures research through March, 2020 and includes research focused on candidate genes and studies that leveraged epigenome-wide data in both children and adults.

## Methods

In accordance with the Preferred Reporting Items for Systematic Reviews and Meta-Analyses (PRISMA) guidelines^21^, we conducted a systematic review of human studies investigating the relationship between childhood maltreatment and other adversities and DNA methylation. For the purposes of this review, childhood was defined as events occurring between birth and age 18. Maltreatment was defined as any experience that involved potential for harm to the child, including emotional, sexual, and physical abuse, and emotional and physical neglect. Other adversities included exposure to intimate partner violence or other violence, early parental death or separation, institutional deprivation, and indentured labor. Empirical articles investigating DNA methylation in association with other forms of childhood stress such as low socioeconomic status or famine, without consideration of other adversities, were not included. Studies focusing on experimentally induced stressors were also not included. In addition, because our focus was on childhood maltreatment after birth; we also did not include studies focusing on prenatal stress or substance use and DNA methylation, which have been addressed in other reviews^22,23^.

Studies were identified by searching PubMed/Medline for empirical articles published in March 2020 or earlier using the following search terms: (((methylation OR epigenetic) AND (child AND (maltreat OR adversity OR trauma OR early life stress OR abuse OR neglect OR ACE))), filtered by Humans. Additional searches in PubMed/Medline for studies published during this timeframe were also conducted using the following search terms: (1) emotion regulation AND methylation; (2) abuse AND methylation NOT substance NOT alcohol; (3) trauma AND methylation; (4) early life stress AND methylation; (5) maltreatment AND methylation; (6) adversity AND methylation. Additional articles published during this timeframe identified through other sources (Google Scholar alerts) were also included.

**Fig. 1:**
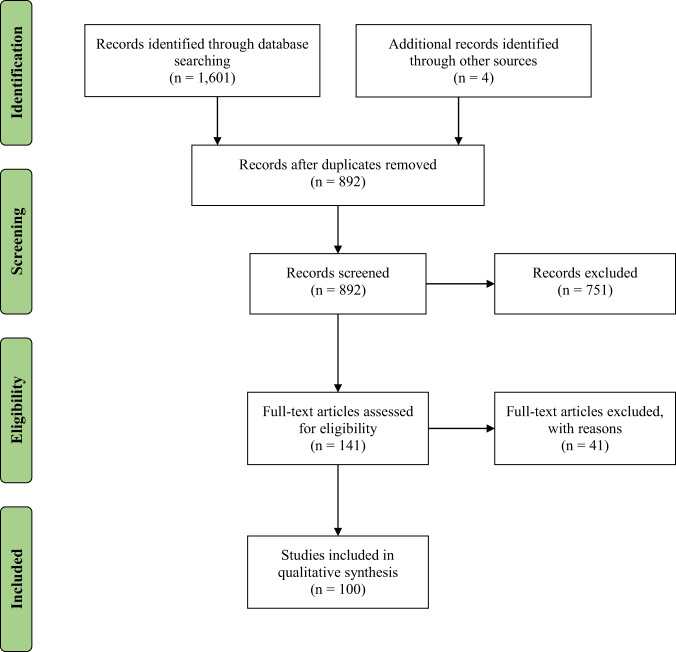
PRISMA flow diagram. Note: Records screened refers to both titles and abstracts.

Our qualitative synthesis included empirical articles that: (1) examined the relationship between childhood maltreatment/adversity and DNA methylation; (2) provided statistical indicators to examine the impact of maltreatment during childhood on DNA methylation; and (3) described maltreatment/adversity that occurred prior to age 18. Empirical articles were excluded if they: (1) did not provide information on childhood maltreatment/adversity and DNA methylation; (2) childhood maltreatment/adversity was not clearly distinguished from adult adversities in study measurement/hypothesis testing; (3) only quality (or quantity) of parental care or support was included as a predictor; (4) were not written in English; (5) were only conducted with animals; and (6) did not use a quantitative approach to summarize research findings (i.e., were qualitative, reviews, comments, or other editorials). We did not omit articles based on small sample size; this information is included in the tables so that it can be considered when evaluating results. Figure 1 documents the methods of our systematic review to generate the final empirical articles included in our qualitative synthesis. Of note, consistent with PRISMA guidelines, records screened includes screening of both titles and abstracts.

## Results

A total of 100 empirical articles were identified in our systematic review focused on the relationship between childhood maltreatment and DNA methylation. This includes 69 empirical articles focused on candidate genes and 31 empirical articles that leveraged epigenome-wide data. Twenty-eight empirical articles measured DNA methylation in childhood and 72 measured DNA methylation in adulthood. Findings are summarized in Tables 1–4, and examples are described below. Major results of each empirical article are included in the tables, though results of specific CpG sites are not detailed given significant design and analytic variability across studies. Given the focus of the current review, only the major study findings related to the association of childhood maltreatment and other adversities with DNA methylation are included in the tables. Empirical articles that focused on another condition (e.g., parental substance use, psychiatric conditions, as described in Sample description in the tables) but included a result pertaining to maltreatment are presented. Importantly, several empirical articles drew upon overlapping samples. We included information regarding the name of the study in the tables if the study name was provided in the empirical article. However, we were unable to denote all articles that may have utilized overlapping samples as the relevant information was not consistently provided in the literature.

**Table 1:** Childhood adversity and candidate gene methylation in samples of children.

Author	Sample	Age(s) at methylation assessment	Gender	Ancestry	Gene(s)	Tissue type	Adversity measurement	Adversity-related findings
Cicchetti & Handley,^26^	*N* = 534; 53% exposed to CM Characterization of sample: Low-income	School age (*M* = 9.4 years)	49% female	61.2% black, 9.9% white, 8.2% biracial or other; 20.6% Latino	*NR3C1*	Saliva	Physical, sexual, emotional abuse, supervisory and physical neglect via child protection case files coded with the MCS	Maltreated children demonstrated hypermethylation compared to non-maltreated children; CM during infancy and/or toddlerhood associated with greater methylation than children with no CM; no difference between children who experienced CM in the preschool period or later and those with no CM; greater chronicity of CM associated with greater methylation; exposure to more CM subtypes associated with greater methylation
Hecker et al.,^29^	*N* = 60; 58% with high exposure to CM; 42% with low exposure to CM Characterization of sample: Participants resided in Tanzania	9–15 years (*M* = 11.3 years in high-exposure group; *M* = 11.8 years in low-exposure group)	60% female in high-exposure group; 56% female in low-exposure group	100% Tanzanian	*NR3C1* *POMC* *CRH* *AVP*	Saliva; blood lymphocytes	Physical and emotional abuse measured via the Maltreatment and Abuse Chronology of Exposure – Pediatric Version, structured interview	Children with high CM exposure had higher methylation of *POMC* and *CRH*, at one CpG site in each gene that survived multiple comparisons correction; differential methylation was also present for several sites in *NR3C1*, *POMC*, and *AVP* but these did not survive multiple comparisons correction
Parade et al.,^12^	*N* = 171; 42% exposed to CM Characterization of sample: Low-income; enrolled in Kids Markers Study; Follow-up of Tyrka, Parade et al. 2015, to examine association of methylation with symptoms/behavior	3–5 years (*M* = 4.2 years)	52% female	23% white non-Hispanic, 48% Hispanic, 15% black, 14% other	*NR3C1*	Saliva	Physical, sexual, emotional abuse, supervisory and physical neglect assessed via child protective services records; death or separation of a caregiver, frequent change of residence or homelessness, inadequate food or clothing, witnessing violence via parent contextual stress interview and Diagnostic Infant and Preschool Assessment	As expected based on prior work with the Kids Markers sample, adversity composite was associated with greater methylation, and CM, past month stress, and lifetime stress were each individually associated with greater methylation whereas traumatic life events were not associated with methylation; new to this analysis, methylation mediated the effect of adversity exposure on internalizing, but not externalizing symptoms
Parent et al.,^13^	*N* = 260; 53% exposed to CM Characterization of sample: Low-income; enrolled in Kids Markers Study; Six-month follow-up of baseline results reported in Tyrka, Parade et al., 2015	3–5 years (*M* = 4.2 years)	52% female	27.7% white non-Hispanic, 45.6% Hispanic, 16.3% black, 21.9% biracial, 2.7% other races	*NR3C1*	Saliva	Physical, sexual, emotional abuse, supervisory and physical neglect assessed via child protective services records; death or separation of a caregiver, frequent change of residence or homelessness, inadequate food or clothing, witnessing violence via parent contextual stress interview and Diagnostic Infant and Preschool Assessment	As expected based on prior work with the baseline data in the Kids Markers sample, CM was associated with higher levels of methylation within 6 months of CM; CM was negatively associated with change in methylation one year after CM, at which point maltreated children demonstrated lower levels of methylation relative to non-maltreated children
Radtke et al.,^142^	*N* = 46 Characterization of sample: Participants resided in Germany	11–21 years (Median = 15 years)	61% female	Not reported	*NR3C1*	Blood lymphocytes	Physical, emotional, and sexual abuse; witnessed violence toward parents, witnessed violence toward siblings, peer physical violence, physical and emotional neglect via a German version of the pediatric Maltreatment and Abuse Chronology of Exposure interview	CM not associated with average methylation; CM was positively associated with methylation at one of 41 examined CpG sites, after adjusting for multiple comparisons using a false discovery rate
Romens et al.,^143^	*N* = 56; 32% with substantiated physical abuse	11–14 years (*M* = 12.1 years)	46% female	66% white, non-Hispanic, 30% black, 4% white, Hispanic	*NR3C1*	Whole blood	Physical abuse via child protective services records	Children with CM had higher levels of methylation at 3 out of 13 CpG sites than children without CM (using a mixed ANOVA method to first test overall interaction of CM and CpG sites [within subjects] followed by planned examination of individual CpG sites when overall interaction was significant)
Tyrka, Parade, et al.,^25^	*N* = 184; 40% exposed to CM Characterization of sample: Low-income; enrolled in Kids Markers Study	3–5 years (*M* = 4.2 years)	51% female	22% white non-Hispanic, 47% Hispanic, 16% black, 15% other races	*NR3C1*	Saliva	Physical, sexual, emotional abuse, supervisory and physical neglect assessed via child protective services records; death or separation of a caregiver, frequent change of residence or homelessness, inadequate food or clothing, witnessing violence via parent contextual stress interview and Diagnostic Infant and Preschool Assessment	An adversity composite, including CM, contextual stress, and traumatic life events, was positively associated with mean methylation; CM and contextual stress were each individually positively associated with methylation
van der Knaap et al.,^144^	*N* = 468 Characterization of sample: Enrolled in the Tracking Adolescents’ Individual Lives Survey	14–18 years (*M* = 16.1 years)	50% female	100% Dutch	*NR3C1*	Whole blood	Perinatal stress assessed using parent interview and record review; traumatic youth experiences assessed using adolescent retrospective self-report; stressful life events assessed using parent interview (childhood events), child self-report (early adolescence events), Event History calendar (middle adolescence events)	Stressful life events and traumatic experiences associated with greater *NR3C1* methylation; Stressful life events in adolescence associated with methylation independent of stressful life events in childhood; Perinatal stress not associated with methylation
Non et al.,^27^	*N* = 117; *n* = 82 institutionalized children, *n* = 35 non-institutionalized children Characterization of sample: Enrolled in Bucharest Early Intervention Project	*M* = 12.5 years	49% female	63% Romanian, 27% Rroma, 9% other	*FKBP5*; *SLC6A4*	Buccal cells	Percentage of time spent in institutional care	Greater time spent in institutional care was associated with lower methylation at one of two CpG sites for *FKBP5* (effect survived multiple comparison correction for analysis within institutionalized group but not in whole sample); Greater time spent in institutional care was associated with lower methylation at two of six CpG sites for *SLC6A4*, significant after multiple comparisons correction; *FKBP5* genotype by adversity interaction was not significant
Parade, Parent, et al.,^107^	*N* = 231; 53% exposed to CM Characterization of sample: Low-income; enrolled in Kids Markers Study; Six-month follow-up of baseline results reported in Tyrka, Ridout, et al., 2015	3–5 years (*M* = 4.3 years)	52% female	40% white, 16% black, 21% biracial, 23% other races	*FKBP5*	Saliva	Physical, sexual, emotional abuse, supervisory and physical neglect assessed via child protective services records; death or separation of a caregiver, frequent change of residence or homelessness, inadequate food or clothing, witnessing violence via parent contextual stress interview and Diagnostic Infant and Preschool Assessment	Maltreated children had lower levels of baseline methylation compared to non-maltreated children, but maltreatment did not predict change in methylation over time; when contextual stress was high, maltreated children had consistently low methylation over time whereas non-maltreated children demonstrated a decline in methylation from baseline to follow-up; *FKBP5* genotype did not moderate associations of CM and methylation
Tyrka, Ridout, et al.,^25^	*N* = 174; 40% exposed to CM Characterization of sample: Low-income; enrolled in Kids Markers Study	3–5 years (*M* = 4.2 years)	52% female	22% white non-Hispanic, 47% Hispanic, 17% black, 15% other races	*FKBP5*	Saliva	Physical, sexual, emotional abuse, supervisory and physical neglect assessed via child protective services records; death or separation of a caregiver, frequent change of residence or homelessness, inadequate food or clothing, witnessing violence via parent contextual stress interview and Diagnostic Infant and Preschool Assessment	Children exposed to CM had lower levels of methylation at two out of two examined CpG sites; there was a trend-level association of lifetime contextual stress and methylation at one site; an adversity composite, including CM, contextual stress, and traumatic life events was negatively associated with methylation at one site; *FKBP5* genotype did not moderate links between adversity exposure and methylation
Timothy et al.,^31^	*N* = 100; *n* = 50 children of alcoholics; *n* = 50 controls, significantly higher adversity in COA group compared to controls Characterization of sample: Resided in urban India	8–16 years (*M* = 11.3 years in COA group; *M* = 11.2 years in control group)	0% female	100% Indian	*SLC6A4*	Saliva	Physical, emotional and sexual abuse, neglect; peer and collective violence; stress of family and friends; pregnancy; problems with alcohol/drugs measured using the WHO Adverse Childhood Experiences Scale	Greater adversity was associated with hypermethylation, particularly in the COA group
van der Knaap et al.,^32^	*N* = 939 Characterization of sample: Enrolled in the Tracking Adolescents’ Individual Lives Survey	14–18 years (*M* = 16.2 years)	51% female	100% Dutch	*SLC6A4*	Whole blood	Perinatal stress assessed using parent interview and record review; traumatic youth experiences assessed using adolescent retrospective self-report; stressful life events assessed using parent interview (childhood events), child self-report (early adolescence events), Event History calendar (middle adolescence events)	More stressful life events associated with higher methylation; effect of stressful life events in adolescence stronger than stressful life events in childhood; *5HTTLPR* genotype moderated effect of stressful life events such that this association was only observed in l-allele homozygotes; perinatal adversity and traumatic youth experiences not associated with methylation
Parade, Novick, et al.,^30^	*N* = 228; 52% exposed to CM Characterization of sample: Low-income; enrolled in Kids Markers Study	3–5 years (*M* = 4.2 years)	53% female	39% white, 17% black, 22% biracial, 22% other races, 45% Hispanic	*HTR2A*	Saliva	Physical, sexual, emotional abuse, supervisory and physical neglect assessed via child protective services records; death or separation of a caregiver, frequent change of residence or homelessness, inadequate food or clothing, witnessing violence via parent contextual stress interview and Diagnostic Infant and Preschool Assessment	CM, traumatic life events, and contextual stress were not associated with methylation; genotype moderated contextual stress to predict methylation, such that contextual stress was positively associated with methylation among A homozygotes, negatively associated among G homozygotes, and not associated among heterozygotes
Barker et al.,^33^	*N* = 785 Characterization of sample: Enrolled in ARIES study nested within ALSPAC	7 years	50% female	100% white	Inflammation-related epigenetic polygenic risk scores (i-ePGS)	Whole blood	Life events (e.g., death in family, accident, and illness), contextual risks (e.g., poor housing conditions and financial problems), parental risks (e.g., parental psychopathology, criminal involvement, and substance use), interpersonal risks (e.g., intimate partner violence and family conflict), and direct victimization (e.g., child bullied by peers or physically hurt) via maternal reports	Postnatal adversity was associated with higher i-ePGS methylation at age 7, which was associated with internalizing symptoms from ages 7–15
Fujisawa et al.,^34^	*N* = 85; 52% exposed to CM Characterization of sample: Those with CM resided in a child welfare facility; controls were recruited from the community.	6–20 years (*M* = 12.9 years)	35% female	100% Japanese	*OXTR*	Saliva	Physical, emotional, sexual abuse, and/or neglect, as determined by the local child welfare facilities	Children who were exposed to CM had higher methylation than children not exposed to CM; methylation was negatively correlated with gray matter volume in the left orbitofrontal cortex; children exposed to CM showed lower gray matter volume compared to the non-CM children

*CM* childhood maltreatment, *MCS* Maltreatment Classification System, *COA* children of alcoholics, *ALSPAC* Avon Longitudinal Study of Parents and Children.

**Table 2:** Childhood adversity and candidate gene methylation in adult samples.

Author	Sample	Age(s) at methylation assessment	Gender	Ancestry	Gene(s)	Tissue type	Adversity measurement	Adversity-related findings
Alexander et al.,^42^	*N* = 200; 49% exposed to CM Characterization of sample: Participants resided in Germany	18–30 years (*M* = 23.7 years)	50% female	100% white	*NR3C1*	Whole blood	Retrospectively assessed physical, sexual, emotional abuse, emotional and physical neglect via the CTQ—Short Form	No difference in mean methylation between CM and control group; methylation moderated effects of CM on cortisol in response to the Trier Social Stress Test; no association of CTQ score and methylation
Barker et al.,^145^	*N* = 94 outpatients with schizophrenia or schizoaffective disorder Characterization of sample: Participants resided in South East Scotland	18–65 years (*M* = 45.8 years)	33% female	Not reported	*NR3C1* *BDNF* *OXTR*	Whole blood	Retrospectively assessed physical, sexual, emotional abuse, emotional and physical neglect via the CTQ	After controlling for multiple comparisons, sexual abuse was associated with methylation at 1 of 25 examined *NR3C1* loci and emotional abuse was associated with methylation at 1 of 73 examined *BDNF* loci; CM not associated with *OXTR* methylation
Bustamante et al.,^38^	*N* = 152; 50% exposed to CM Characterization of sample: Enrolled in Detroit Neighborhood Health Study	*M* = 49.6 years	62% female	76% African American; 17% European American; 7% other	*NR3C1*	Whole blood	Retrospectively assessed physical, sexual, and emotion abuse via the Conflict Tactics Scale and CTQ	Participants with CM exposure showed increased average methylation across four CpG sites, after Benjamini Liu False Discovery Rate correction
Farrell et al.,^52^	*N* = 67, *n* = 33 depressed patients, *n* = 34 controls Characterization of sample: Participants resided in Ireland	**≤** 45 years; (*M* = 28.3, patients; *M* = 28.2, controls)	67% female	Not reported	*NR3C1* *FKBP5*	Whole blood	Retrospectively assessed physical, sexual, emotional abuse, emotional and physical neglect via the CTQ	Greater severity of emotional abuse was associated with higher *NR3C1* methylation in depressed patients at two of five CpG sites; no associations with other forms of CM; no associations between CM and *FKBP5*
Fiacco et al.,^146^	*N* = 103; 71% exposed to CM Characterization of sample: Enrolled in the Women 40+ Health Aging Study; participants resided in Zurich	40–73 years (*M* = 53.4 years)	100% female	Not reported	*NR3C1* *ERα*	Dried capillary blood spots	Retrospectively assessed physical, sexual, emotional abuse, emotional and physical neglect via the German version of the CTQ	CM not associated with percent *NR3C1* methylation; after controlling for multiple testing, participants with emotional abuse had higher percent methylation in *ERα* shore than those without, and higher levels of CM were also associated with higher levels of *ERα* shore methylation
Labonté, Yerko, et al., 2012a^37^	*N* = 56; *n* = 21 in CM/suicide group, *n* = 21 in non-CM/suicide group, *n* = 14 controls without suicide or CM Characterization of sample: Quebec Suicide Brain Bank	*M* = 37 years in CM/suicide group; *M* = 40.8 years in non-CM suicide group; *M* = 39.8 years in control group	0% female	100% Caucasian of French-Canadian descent	*NR3C1*	Brain (Hippocampus, Anterior Cingulate Cortex (ACC))	Retrospectively assessed sexual abuse, physical abuse and severe neglect via psychological autopsy, including structured interviews/chart reviews using the CECA adapted for psychological autopsies	*NR3C1* gene expression reduced in the CM/suicide group across hippocampus and within alternate first exons; no expression differences in ACC, methylation analyses focused on hippocampus. No differences in total % methylation across all CpG sites between groups in hippocampus; differential methylation patterns were observed at different alternate first exons. At exon 1_B_ there was a group by CpG interaction, with increased and decreased methylation at different CpG sites in both suicide groups relative to controls. At exon 1 _C_ the group by CpG site interaction was not significant. At exon 1_H_ there was a main effect of group, with significant hypomethylation in the CM/suicide group compared with the other two groups. In contrast to 1B and 1 C promoters, 1H promoter methylation was positively associated with gene expression
Martín-Blanco et al.,^39^	*N* = 281 patients with borderline personality disorder; 73% exposed to CM Characterization of sample: Participants resided in Spain	*M* = 29.4 years	85% female	100% Caucasian or European descent	*NR3C1*	Whole blood	Physical, sexual, emotional abuse, emotional and physical neglect via the CTQ-SF	Methylation positively associated with CM, specifically physical abuse
McGowan et al.,^36^	*N* = 36; *n* = 12 completed suicide and exposed to CM, *n* = 12 completed suicide but no CM, *n* = 12 controls with sudden accidental death and without CM Characterization of sample: Quebec Suicide Brain Bank	*M* = 34.2 years in CM/suicide group; *M* = 33.8 years in no CM/suicide group; *M* = 35.8 years in control group	0% female	100% Caucasian of French-Canadian descent	*NR3C1*	Brain (hippocampal tissue)	Retrospectively assessed sexual abuse, physical abuse and severe neglect via psychological autopsy, including structured interviews/chart reviews using the CECA adapted for psychological autopsies	CM/suicide group had significantly lower *NR3C1* expression, and greater *NR3C1* methylation across exon 1 _F_ compared to the two other groups
Melas et al.,^35^	*N* = 176 *NR3C1* methylation analysis; *n* = 93 with depression, 70% exposed to childhood adversity; *n* = 83 control, 63% exposed to childhood adversity *N* = 174 MAOA methylation analysis; *n* = 82 with depression, 70% exposed to childhood adversity; *n* = 92 control, 60% exposed to childhood adversity Characterization of sample: Enrolled in the PART study	29–74 years	100% female	100% Swedish nationals	*NR3C1* *MAOA*	Saliva	Retrospectively assessed early parental death, divorce, financial problems or other familial constraints based on self-reported answers provided in the PART study questionnaire	Childhood adversity not associated with *MAOA* methylation; early parental death and familial constraints were associated with overall *NR3C1* hypermethylation; MAOA-L genotype mediated the association of early parental death and methylation
Peng et al.,^59^	Sample 1: *N* = 168 (84 male twin pairs), 57% exposed to CM Sample 2: *N* = 70 (35 female twin pairs), 43% exposed to CM Characterization of samples: Sample 1: Enrolled in Twins Heart Study (THS); Sample 2: Enrolled in Mood and Methylation Study	Sample 1: *M* = 55 years; Sample 2: *M* = 36 years	29% female	Not reported	*NR3C1* *BDNF* *MAOA* *MAOB* *SLC6A4*	Sample 1: Whole blood; Sample 2: Blood monocytes	Retrospectively assessed physical, sexual, emotional abuse and general trauma via the Early Trauma Inventory Self Report—Short Form	Sample 1: Associations (q < 0.10) of exposure to CM/childhood trauma and hypermethylation of individual CpG sites of *BDNF*, *NR3C1*, *MAOB*, and *SLC6A4* after correcting for multiple comparisons Sample 2: Emotional abuse associated with hypermethylation of *NR3C1*
Perroud et al.,^40^	*N* = 215; *n* = 101 subjects with borderline personality disorder, 95% exposed to CM; *n* = 99 subjects with psychiatric disorders, 50% exposed to CM; *n* = 15 depressed subjects with history of PTSD, 100% exposed to CM Characterization of sample: Participants resided in France and Switzerland	*M* = 36.22 years	79% female	Not reported	*NR3C1*	Whole blood	Physical, sexual, emotional abuse, emotional and physical neglect via the CTQ	CM associated with higher methylation compared to those with no CM; number of types of CM positively correlated with methylation
Schür et al.,^43^	*N* = 241; *n* = 131 healthy controls, *n* = 13 patients with schizophrenia, *n* = 43 patients with bipolar disorder; and *n* = 54 unaffected siblings of patients with schizophrenia and bipolar disorder Characterization of sample: Participants resided in Netherlands/UK	*M* = 37.4 years	40% female	Not reported	*NR3C1*	Whole blood	Retrospectively assessed physical, sexual, emotional abuse, emotional and physical neglect via the CTQ	No association between CM and methylation
Shields et al.,^147^	*N* = 295; 52% exposed to CM Characterization of sample: Enrolled in Black Women’s Health Study	43–78 years (Median = 53 years)	100% female	100% African American	*NR3C1*	Whole blood	Retrospectively assessed physical and sexual abuse via a 9-item instrument adapted from the Conflict Tactics Scale and the Pregnancy Abuse Assessment Screen	There was no significant difference in methylation between women with and without CM; women with the highest physical abuse frequency did show significantly greater mean percent methylation compared to women without physical abuse; women with 1–3 incidents of sexual abuse demonstrated greater methylation than non-sexually abused counterparts; emotional support moderated associations among women with the highest levels of physical and sexual abuse
Steiger et al.,^44^	*N* = 96; *n* = 32 with bulimia nervosa and extreme CM, *n* = 32 with BN and no CM, *n* = 32 women without BN or CM	17–48 years (*M* = 26.05 years in BN group; *M* = 23.67 years in non-BN group)	100% female	Not reported	*NR3C1*	Whole blood	Retrospectively assessed sexual or physical abuse via the Childhood Trauma Interview	No significant differences between CM-exposed group and nonexposed group
Suderman et al.,^148^	*N* = 24; *n* = 12 suicide completers exposed to severe CM, *n* = 12 controls with no CM Characterization of sample: Quebec Suicide Brain Bank	Postmortem	100% male	French-Canadian origin	*NR3C1*	Hippocampal tissue	Severe sexual and/ or physical abuse or severe neglect, as determined by most severe scores in the structured CECA questionnaire adapted for psychological autopsies	CM associated with differential methylation, with both increased (155 DMRs) and decreased (126 DMRs) methylation across the *NR3C1* locus
Tyrka et al.,^11^	*N* = 99	18–59 years (*M* = 27.3 years)	59% female	Not reported	*NR3C1*	Whole blood	Retrospectively assessed physical, sexual, emotional abuse, emotional and physical neglect via the CTQ; loss of a parent via two items	Adversity index, encompassing CM, parental loss, and parental care was positively correlated with methylation at two sites; CM and parental loss individually associated with one or two CpG sites, respectively
Tyrka et al.,^41^	*N* = 340 (57% exposed to adversity)	18–65 years *M* = 32.9 years)	63% female	78% white	*NR3C1*	Whole blood	Retrospectively assessed physical, sexual, emotional abuse, emotional and physical neglect via the CTQ, and death or separation from a parent via participant interview	Those with adversity/no mental health disorder and adversity/mental health disorder had lower levels of mean methylation than the no adversity/no disorder group; each adversity type was associated with lower levels of methylation, and the number of adversities was negatively associated with methylation only in participants with no mental health disorder
Vangeel et al.,^45^	*N* = 95; *n* = 76 in chronic fatigue group, 39% with moderate/severe childhood trauma; *n* = 19 in control group with no trauma history	*M* = 44.2 years	100% female	100% white	*NR3C1*	Whole blood	Retrospectively assessed physical and sexual abuse in 27 participants using the Structured Trauma Interview (Dutch version); retrospectively assessed emotional neglect, emotional abuse, physical abuse, sexual harassment, and sexual abuse in 49 participants using the Traumatic Experiences Checklist and review of medical records	No associations of childhood trauma and methylation
Wang et al.,^46^	*N* = 149; *n* = 64 diagnosed with generalized anxiety disorder (GAD), *n* = 85 healthy controls; 11% of total sample exposed to CM Characterization of sample: Participants resided in China	*M* = 35.4 years in GAD group; *M* = 33.9 years in control group	68% female	97% Chinese Han	*NR3C1*	Peripheral blood mononuclear cells	Retrospectively assessed physical, sexual, emotional abuse, emotional and physical neglect via the CTQ—Short Form	CM (present in 11% of the sample) not associated with methylation
Bustamante et al.,^50^	*N* = 112, 50% exposed to CM Characterization of sample: Enrolled in Detroit Neighborhood Health Study	*M* = 50.7 years	55% female	77% African American, 16% European American, 7% other	*FKBP5*	Whole blood	Retrospectively assessed physical, sexual, and emotion abuse via the Conflict Tactics Scale and CTQ	No association between CM and methylation; *FKBP5* genotype did not moderate nonsignificant association of CM and methylation in a subset of the participants with genotype data available (*n* = 100)
Harms et al.,^49^	*N* = 54; 54% with high levels of stress	19–23 years (*M* = 20.5 years)	52% female	59% white, 28% African American, 5% Hispanic, 8% Asian	*FKBP5*	Saliva	Prospectively assessed stressful life events via the Youth Life Stress Interview	Stressful life events in childhood, but not adulthood, were positively correlated with methylation; association of early life stress with inhibition-related prefrontal activity was mediated by methylation
Klengel et al.,^47^	Sample 1: Grady Trauma Project subsample, *N* = 76; 39% with high CM, 61% with no CM Sample 2: Conte Center subsample, *N* = 56; 100% with CM Characterization of sample: GTP subsample: urban, low-income Conte Center subsample: urban	GTP: *M* = 41.5 years in CM group; *M* = 41.0 years in control group Conte: *M* = 28.5 years	GTP:76% female Conte: 100% female	GTP:95% African American 3% Caucasian 1% Mixed 1% Other Conte: 55% African American 30% Caucasian 4% Mixed 11% Other	*FKBP5*	Saliva; Whole blood	Retrospectively assessed physical and sexual abuse via the CTQ and lifetime history of trauma via the Traumatic Events Inventory	CM associated with lower methylation among adults with rs1360780 T risk allele (this result observed in both GTP and Conte Center samples)
Klinger-König et al.,^51^	*N* = 3965, low rates of CM Characterization of sample: Participants resided in Germany, Enrolled in the Study of Health in Pomerania	20–79 years (*M* = 54.2 years)	52% female	100% Caucasian	*FKBP5*	Whole blood	Retrospectively assessed physical, sexual, emotional abuse, emotional and physical neglect via the CTQ	No association between CM and methylation, and *FKBP5* genotype did not moderate nonsignificant associations
Tozzi et al.,^48^	*N* = 106; *n* = 55 depressed patients, *n* = 50 controls Characterization of sample: Participants resided in Ireland	18–65 years *M* age ranged from 34 years to 40.4 years across subsamples	64% female in depressed group; 60% female in control group	Not reported	*FKBP5*	Whole blood	Retrospectively assessed physical, sexual, emotional abuse, emotional and physical neglect via the CTQ	Higher CM associated with lower methylation in the high-risk T-allele group with depression
Yeo et al.,^53^	*N* = 29, 55% with high CM and substance dependence	*M* = 45.2 years as reported in Roy et al., 2010	100% male	100% African American	*FKBP5*	Blood lymphoblast cell lines	Retrospectively assessed physical, sexual, emotional abuse, emotional and physical neglect via the CTQ	No association between CM and methylation
Pape et al.,^149^	*N* = 88 patients with PTSD, *n* = 43 treated with GSK561679, *n* = 45 placebo; 65% of sample exposed to CM	18–65 years	100% female	Not reported	*CRHR1*	Whole blood	Retrospectively assessed physical, sexual, emotional abuse, emotional and physical neglect via the CTQ	CM not associated with baseline *CRHR1* methylation; CM interacted with rs110402 genotype to predict change in *CRHR1* methylation following GSK561679 treatment
Beach et al.,^54^	*N* = 192 Characterization of sample: Enrolled in Iowa Adoption Study	35–69 years	50% female	Not reported	*SLC6A4*	Blood lymphoblast cell lines	Retrospectively assessed child physical or sexual abuse via questions eliciting presence or absence of abuse	Physical and sexual abuse in childhood associated with greater overall methylation of *SLC6A4* promoter region for both males and females
Beach et al.,^55^	*N* = 155; 13% exposed to sexual abuse Characterization of sample: Enrolled in Iowa Adoption Study	*M* = 41.8 years	100% female	Not reported	*SLC6A4*	Blood lymphoblast cell lines	Retrospectively assessed child sexual abuse via questions eliciting presence or absence of abuse	Sexual abuse in childhood associated with methylation of the *5HTT* promoter region
Beach et al.,^56^	*N* = 155; 17% exposed to a broader measure of sexual abuse than in Beach et al.^55^ Characterization of sample: Enrolled in Iowa Adoption Study	*M* = 41.1 years	100% female	94% white	*SLC6A4*	Blood lymphoblast cell lines	Retrospectively assessed child sexual abuse via questions eliciting presence or absence of abuse	Sexual abuse in childhood positively associated with methylation; interaction of genetic load × sexual abuse predicted methylation––main effect of sexual abuse on methylation for those with greater genetic load was strong, but marginal for those with no genetic load
Booij et al.,^57^	*N* = 69*; n* = 33 patients with depression, *n* = 36 controls; 52% (depressed + control) exposed to CM Characterization of sample: Participants resided in Ireland	18–65 years	64% female	Not reported	*SLC6A4*	Whole blood	Retrospectively assessed physical, sexual, emotional abuse, emotional and physical neglect via the CTQ	CM associated with methylation across *SLC6A4* promoter region; physical abuse, but not other types of abuse/neglect associated with methylation; 5-HTTPR *ll* genotype × CM interaction demonstrated greater *SLC6A4* methylation relative to *s*-carriers with or without CM
Kang et al.,^58^	*N* = 102 patients with depression; 33% exposed to adversity Characterization of sample: Participants resided in Korea	*M* = 54.9 years	75% female	Not reported	*SLC6A4*	Whole blood	Retrospectively assessed parental loss, financial hardship, physical abuse, and sexual abuse	Any adversity, parental loss, physical abuse, and sexual abuse were associated with higher methylation average percentage
Okada et al.,^60^	*n* = 50 patients with depression Characterization of sample: Participants resided in Japan	21–62 years (*M* = 40.3 years)	46% female	100% Japanese	*SLC6A4*	Whole blood	Retrospectively assessed early adversity via Early Trauma Inventory Self Report-Short Form	Early adversity was negatively correlated with one CpG site, and positively correlated with another site, of a total of 29 CpG sites and significance set at P < 0.05
Wankerl et al.,^61^	*N* = 133; 13% with CM Characterization of sample: Participants resided in Germany	18–30 years (*M* = 23.8 years)	47% female	100% Caucasian	*SLC6A4*	Whole blood	Retrospectively assessed physical, sexual, emotional abuse, emotional and physical neglect using the CTQ—Short Form	No significant association of CM and mean methylation
Wang, Lv, et al.,^150^	*N* = 85 patients with depression Characterization of sample: Participants resided in China	*M* = 36.7 years	66% female	100% Chinese Han	*HTR1A* *HTR1B*	Whole blood	Retrospectively assessed physical, sexual, emotional abuse, emotional and physical neglect via the CTQ	No association between CM and mean methylation of *5HTR1A* or *5HTR1B*; CM inversely correlated with methylation at two of 96 CpG sites within *5HTRB*, after correcting for multiple comparisons
Perroud et al.,^151^	*N* = 346; *n* = 122 patients with bipolar disorder, *n* = 116 with borderline personality disorder, and *n* = 111 with attention deficit hyperactivity disorder Characterization of sample: Participants resided in Switzerland	*M* = 45.3 years in BD group, *M* = 31.5 years in BPD group, *M* = 37.7 years in ADHD group	BD: 53% female; BPD: 91% female, ADHD: 30% female	100% European ancestry	*5-HT3AR*	Whole blood	Retrospectively assessed physical, sexual, emotional abuse, emotional and physical neglect via the CTQ	Physical abuse was positively associated with methylation at two of 11 CpG sites and negatively associated with methylation at one of 11 CpG sites after adjusting for multiple comparisons; CTQ total score and other CM types not associated with individual CpG sites after adjusting for multiple comparisons; patients with highest severity of CM and CC genotype of rs1062613 had the highest methylation; methylation at two CpG cites mediated association of physical abuse and history of suicide attempt, previous hospitalization, and number of mood episodes
Checknita et al.,^152^	*N* = 194; *n* = 131 patients from adolescent substance abuse clinic, *n* = 40 siblings of clinic patients, *n* = 23 healthy controls 51% exposed to CM Characterization of sample: Participants resided in Sweden	*M* = 22 years	60% female	Not reported	*MAOA*	Saliva	Retrospectively assessed physical abuse via the Conflict Tactics Scales and sexual abuse via the Sexual Experience Survey, Sexual and Physical Abuse Questionnaire, and McArthur Community Violence Instrument	Among females, CM was associated with higher methylation for one of three components of the region of interest of *MAOA*; among males, no differences in methylation of ROI between those with and without CM
Gouin et al.,^153^	*N* = 46, 52% with high CM Characterization of sample: Participants resided in Québec	27 years	50% female	100% Caucasian of Western European ancestry	*OXTR*	Whole blood	Retrospectively assessed physical abuse using Parent-Child Conflict Tactics Scale and sexual abuse using adaptations of Adverse Childhood Experiences Questionnaire and the Sexually Victimized Children Questionnaire	No overall methylation difference between high and low CM groups; among females, the high CM group had significantly higher methylation at one of 16 CpG sites after adjusting for multiple comparisons
Kogan et al.,^154^	*N* = 358 Characterization of sample: Participants resided in rural South Georgia	*M* = 21.9 years	100% male	100% African American	*OXTR*	Saliva	Retrospectively assessed physical, sexual, emotional abuse, emotional and physical neglect via the CTQ—Short Form	No association between CM and methylation; CM indirectly associated with contemporary prosocial ties, which predicted elevated methylation
Kogan et al.,^94^	*N* = 309 Characterization of sample: Participants resided in rural South Georgia	*M* = 21.9 years	100% male	100% African American	*OXTR*	Saliva	Retrospectively assessed physical, sexual, emotional abuse, emotional and physical neglect via the CTQ—Short Form	No association between CM and methylation; CM positively associated with socioeconomic instability, which predicted elevated methylation
Smearman et al.,^63^	*N* = 393, 49% exposed to CM Characterization of sample: Low-income, urban	18–77 years (*M* = 41 years)	71% female	100% African American	*OXTR*	Whole blood	Retrospectively assessed physical, sexual, emotional abuse, emotional and physical neglect via the CTQ	No association between CM and methylation; methylation did not mediate association between CM and psychiatric symptoms; CM and methylation interacted to predict depression and anxiety at three CpG sites
Womersley et al.,^155^	*N* = 63; 59% with emotional neglect; 70% of sample diagnosed with social anxiety disorder (many with comorbid or other diagnoses) Characterization of sample: Participants resided in South Africa	25–41 years (Median = 31 years)	56% female	100% Caucasian	*OXTR*	Whole blood	Retrospectively assessed emotional neglect via the CTQ—Short Form	No association between emotional neglect and *OXTR* methylation
Janusek et al.,^64^	*N* = 34 Characterization of sample: Participants resided in Chicago metropolitan low-income neighborhoods	18–25 years (*M* = 20.2 years)	100% male	100% African American	*IL-6*	Peripheral blood mononuclear cells	Retrospectively assessed emotional, physical, and sexual abuse, emotional and physical neglect, parental substance abuse, parental mental illness, violent treatment of mother or stepmother, parental separation or divorce, and criminal behavior in the household via the CTQ and ACE study questionnaire	Increased CM associated with reduced methylation
Perroud et al.,^156^	*N* = 167; *n* = 115 borderline personality disorder group, *n* = 52 controls recruited from the School of Dentistry at the University of Geneva	*M* = 33.6 years	79% female	Not reported	*BDNF*	Whole blood	Physical, sexual, emotional abuse, emotional and physical neglect via the CTQ	Methylation was associated with greater number of childhood traumas
Thaler et al.,^157^	*N* = 96; *n* = 32 with bulimia nervosa and extreme CM, *n* = 32 with BN and no CM; *n* = 32 women without BN or CM	17–48 years (*M* = 26.1 years for BN groups; *M* = 23.7 years for non-BN group)	100% female	Not reported	*BDNF*	Whole blood	Retrospectively assessed sexual and physical abuse via the Childhood Trauma Interview	Participants with BN and physical abuse had greater percentage of methylation than controls across all CpG sites, with five of 30 examined sites demonstrating significantly greater methylation; participants with BN and sexual abuse had greater percentage of methylation at one of 30 examined sites
Wang, Zhang, et al.,^158^	*N* = 85 patients with depression Characterization of sample: Participants resided in China	*M* = 36.7 years	66% female	100% Chinese Han	*BDNF*	Whole blood	Retrospectively assessed physical, sexual, emotional abuse, emotional and physical neglect via the CTQ	CM negatively associated with mean methylation
Boks et al.,^159^	*N* = 93 military personnel; *N* = 85 controls Characterization of sample: Participants resided in the Netherlands	*M* = 27.5 years for military group; *M* = 33 years for control group	0% female in military group; 51% female in control group	100% European Caucasian	*SKA2*	Whole blood	Military: Retrospectively assessed general trauma, physical abuse, emotional abuse, and sexual abuse via the Early Trauma Inventory-Self Report Control: Retrospectively assessed physical, sexual, emotional abuse, emotional and physical neglect via the CTQ	CM not associated with prospective changes in methylation in military group; methylation did not mediate association between CM and cortisol reactivity in control group
He et al.,^160^	*N* = 141; *n* = 50 patients with bipolar disorder, *n* = 91 healthy controls	*M* = 43.5 years in bipolar group; *M* = 33.5 years in control group	49% female	100% Caucasian, with 3 or more Dutch grandparents	*KITLG*	Whole blood	Retrospectively assessed physical, sexual, emotional abuse, emotional and physical neglect via the CTQ	CM was positively associated with methylation in the control group, but not in the bipolar disorder group
Wrigglesworth et al.,^161^	*N* = 142; 15% exposed to physical or sexual abuse, 20% exposed to parental death Characterization of sample: Enrolled in the ESPRIT study; resided in France	65–80 years (*M* = 69.7 years)	49% female	Not reported	*KITLG*	Whole blood	Retrospectively assessed 25 adverse events including physical abuse, sexual abuse, and parental death via the Childhood Adversity Questionnaire	No association between childhood adversity and methylation
Berent et al.,^162^	*N* = 303; *n* = 176 patients with alcohol use disorder, *n* = 127 healthy controls Characterization of sample: Participants were native inhabitants of Poland	*M* = 43.4 years in alcohol use disorder group; *M* = 39.5 years in control group	24% female	100% Polish Caucasian	*SSTR4*	Buccal cells	Retrospectively assessed 13 ACEs: physical, verbal, and sexual abuse, neglect, loss of parent, domestic violence, parental mental illness, parental alcohol abuse, parental drug abuse, parental incarceration, witnessing family member’s suicide attempt, witnessing family member’s death due to any cause, and witnessing stranger’s death due to any cause	No association between adversity and methylation
Lutz, Gross, et al.,^84^	*N* = 94; *n* = 60 depressed adults who died by suicide, 50% with severe CM; *n* = 34 psychiatrically healthy controls with sudden accidental death Characterization of sample: Douglas Bell Canada Brain Bank	Postmortem; *M* = 43.4 years in suicide with CM group; *M* = 47.1 years in suicide without CM group; *M* = 46.3 years in control group	18% female	Not reported	*Kappa*	Brain (Anterior insula tissue)	Retrospectively assessed severe physical, sexual abuse and neglect via adapted version of CEMA interview, validated using medical charts, coroner files, and reports from child protective services	CM associated with decreased DNA methylation in one of six examined genomic regions of *Kappa*, and selective reduction of DNA hydroxymethylation, as well as decreased *Kappa* gene expression
Thomas et al.,^163^	Tuebingen Cohort: *N* = 151 patients with depression and healthy controls; 58% exposed to CM Characterization of sample: Participants resided Germany PReDICT Cohort: *N* = 299 patients with depression; 66% exposed to CM Characterization of sample: Enrolled in the PReDICT project Grady Cohort: *N* = 310; 51% exposed to CM Characterization of sample: Low-income; enrolled in Grady Trauma Project	Tuebingen Cohort: *M* = 32.7 years PReDICT Cohort: *M* = 40.2 years Grady Cohort: *M* = 42.0 years	Tuebingen Cohort: 67% female PReDICT Cohort: 58% female Grady Cohort: 26% female	Tuebingen Cohort: 100% Caucasian PReDICT Cohort: Not reported Grady Cohort: Not reported	*MORC1*	Whole blood	Retrospectively assessed physical, sexual, emotional abuse, emotional and physical neglect via the CTQ	CM not associated with mean methylation in any cohort, nor methylation at any individual CpG site (12 sites assessed in PReDICT and Grady, 50 in Tuebingen)
Lapp,^164^	*N* = 90; 37% with 1–3 ACEs, 29% with 4 or more ACEs Characterization of sample: Participants recruited from University of Massachusetts and the greater Boston area	*M* = 32.1 years	48% female	51% white, 22% African American, 14% Asian, 7% Hispanic, 6% other/multiple	*MT-ND6*	Buccal cells	Retrospectively assessed ACEs using the Adverse Childhood Experiences Survey	Lower levels of mean methylation in individuals with no ACEs compared to individuals with four or more ACEs
Misiak et al.,^165^	*N* = 96; *n* = 48 patients with first episode schizophrenia, *n* = 48 healthy controls; 38% of first episode schizophrenia patients and 4% of healthy controls exposed to childhood trauma Characterization of sample: Participants resided in Poland	*M* = 26 years	54% female	Not reported	*BAGE (B-melanoma antigen)* *LINE-1*	Whole blood	Retrospectively assessed general trauma, physical punishment, emotional and sexual abuse via Early Trauma Inventory Self Report –Short Form	First episode schizophrenia patients with CM demonstrated significantly less *LINE-1* methylation compared with first episode schizophrenia patients and healthy controls without CM; CM was not predictive of *LINE-1* methylation in healthy controls; there were no significant differences in *BAGE* methylation between first episode schizophrenia patients with/without CM and healthy controls without CM; higher general trauma score predicted lower *BAGE* methylation and trend associations of *BAGE* methylation, physical punishment, and total trauma in healthy controls

*CM* childhood maltreatment, *CTQ* Childhood Trauma Questionnaire, *CECA* Childhood Experience of Care and Abuse, *DMR* differentially methylated region, *FDR* False Discovery Rate, *BN* bulimia nervosa, *GTP* Grady Trauma Project, *BD* bipolar disorder, *BPD* borderline personality disorder, *ADHD* attention deficit hyperactivity disorder, *PTSD* post-traumatic stress disorder.

**Table 3:** Childhood adversity and studies leveraging epigenome-wide methylation arrays in samples of children.

Author	Sample	Age(s) at methylation assessment	Gender	Ancestry	Tissue type	Adversity measurement	Adversity-related findings
Cecil et al.,^166^	*N* = 124; 68% exposed to CM Characterization of sample: Inner city youth in London	16–24 years	53% female	49% white, 33% black, 18% other	Buccal cells	Physical, sexual, emotional abuse, emotional and physical neglect via the CTQ	Physical and sexual abuse, and physical neglect associated with epigenetic variation; no significant association between emotional abuse and neglect and epigenetic variation; top loci (e.g., *GABBR1, GRIN2D, CACNA2D4, PSEN2)* include markers implicated in stress-sensitive outcomes; gene ontology supports some common epigenetic signatures related to growth and neural development
Cicchetti et al.,^78^	*N* = 548; 54% exposed to CM Characterization of sample: Low-income	School age (*M* = 9 years)	48% female	68% black, 21% white, 12% biracial or other; 21% Latino	Saliva	Physical, sexual, emotional abuse, supervisory and physical neglect via child protection case files coded with the MCS	Maltreated children had higher levels of methylation at sites where methylation was generally low, and lower methylation at sites where methylation generally high, compared to non-maltreated children; Additional site-specific analyses using a candidate approach examined *AKDH2, ANKK1*, and *NR3C1*; maltreated girls had lower methylation of *ALDH2* than non-maltreated girls, and maltreated boys had higher *ALDH2* methylation than non-maltreated boys; boys with early, but not recent, maltreatment had higher methylation of *ALDH2* than non-maltreated boys; early maltreated children also had higher *ANKK1* methylation than non-maltreated children
Dunn et al.,^79^	*N* = 774; 67% exposed to adversity Characterization of sample: Enrolled in ARIES study nested within ALSPAC	7 years	50% female	Not reported	Cord blood at birth and peripheral blood at age 7	Physical, sexual, emotional abuse; maternal psychopathology; single parent; family instability; financial stress/poverty; neighborhood disadvantage measured via maternal report from a single item or from psychometrically validated standardized measures	Nearly all types of adversity exposure in early childhood (age 3 or younger) associated with methylation differences at age 7; only physical and sexual abuse in middle childhood associated with methylation differences at age 7; accumulation and recency of adversity did not explain variance in methylation; findings supported gene ontology pathways involving growth, axon development, and neuron apoptotic processes
Esposito et al.,^167^	*N* = 83; 60% institutionalized in Eastern Europe or Russia and adopted in the U.S.; 40% raised with biological families in the U.S.	12–18 years in adopted group (*M* = 15.7 years); 13–17 years in non-adopted group (*M* = 15.4 years)	52% female	100% European descent	Peripheral blood mononuclear cells	46 stressful life events within the past year via the Life Events Checklist—Child/Adolescent version; history of institutionalization	Children adopted from institutions of abandoned or orphaned children in Eastern Europe or Russia had higher levels of methylation compared to children of similar ancestry raised by biological parents in the United States; although after correction for cell type and multiple covariates, adoption groups were not associated with methylation, observed leftward skew supported a separate step of filtration to remove sites with no variation and genes with differentially methylated sites included *TMEM200C, PPP1R3G, GLYATL2, CYP1A1*, and *miR-324*.; however differential methylation in *CYP1A1* may be due to adopted children having had higher early life exposures to cigarette smoke, or current smoking
Jovanovic et al.,^81^	*N* = 101	6–13 years (*M* = 9.7 years)	55% female	100% African American	Saliva	9 items capturing direct violence exposure and 13 items measuring witnessing violent events via the Violence Exposure Scale for Children-Revised, a cartoon-based self-report interview of children’s lifetime exposure to violence	Exposure to direct violence was positively associated with epigenetic age acceleration (the residual between DNA methylation age and chronological age); the group with the most age acceleration had the most trauma
Kumsta et al.,^168^	*N* = 49; *n* = 16 with extended institutional deprivation; *n* = 17 with limited institutional deprivation; *n* = 16 control with no institutional deprivation Characterization of sample: Enrolled in ERA study; children adopted from Romania into families residing in England	11–15 years	Not reported	100% Romanian in institutional deprivation groups; 100% UK nationals in control group	Buccal cells	Duration of institutional deprivation categorized as none, less than, or greater than six months	Children exposed to more than 6 months in Romanian institutions (extended institutional deprivation) showed elevated methylation; methylation of early adopted Romanian children (limited institutional deprivation) was not significantly different from control group with no institutional deprivation
Marini et al.,^169^	*N* = 973; 13% exposed to physical abuse, 49% exposed to family instability Characterization of sample: Enrolled in ALSPAC	7.5 years	50% female	97% white	Whole blood	Physical, sexual, emotional abuse; maternal psychopathology; single parent; family instability; financial stress/poverty; neighborhood disadvantage measured via maternal report from a single item or from psychometrically validated standardized measures	Financial hardship associated with epigenetic age acceleration; using Hannum’s epigenetic clock, sexual or physical abuse at 3.5 years associated with older epigenetic age, financial hardship and neighborhood disadvantage at 7 years associated with acceleration in epigenetic aging; no associations emerged using Horvath’s epigenetic clock
Naumova et al.,^170^	*N* = 28; *n* = 14 raised since birth in institutional care, *n* = 14 raised by biological parents Characterization of sample: Participants resided in northwest region of the Russian Federation	7–10 years (*M* = 8.1 years in institutionalized group; *M* = 8.4 years in control group)	36% female in institutionalized group; 29% female in control group	Predominantly Slavic	Whole blood	General trauma, physical punishment, emotional and sexual abuse prior to age 18 assessed using the Early Trauma Inventory Self Report-Short Form	The institutionalized group demonstrated overall proportionally greater methylation relative to the controls; analysis of pathway enrichment supported enrichment of the upmethylated (increased methylation) genomes of institutionalized children in regions related to cellular signaling and immune response; functional annotation implicated methylation profile differences in genes related to brain function (including genes in the dopaminergic system, glucocorticoid and steroid biosynthesis, serotonergic system, etc.)
Papale et al.,^171^	*N* = 22; 50% exposed to high stress	9–12 years (*M* = 10.9 years)	100% female	50% Caucasian	Saliva	Life stress measured by the Youth Life Stress Interview	High stress exposure was associated with variability in methylation
Sumner et al.,^80^	*N* = 247 Characterization of sample: 27% below poverty line	8–16 years (*M* = 12.7 years)	48% female	39% white, 28% black, 21% other, 12% Hispanic	Saliva	Physical, sexual, and emotional abuse; domestic violence exposure; exposure to other interpersonal violence; emotional neglect; food insecurity via child interview and self-report measures	Exposure to threat associated with accelerated DNA methylation age and advanced pubertal stage, but exposure to deprivation was not; threat exposure affected depressive symptoms through DNA methylation age
Weder et al.,^172^	*N* = 190; 50% exposed to CM	5–14 years (*M* = 10.2 years)	58% female	17% European American, 38% Hispanic, 30% African American, 15% biracial	Saliva	CM assessed via child protection records, parent and child reports of trauma using the KSADS interview, child reports on the CTQ, maternal reports of domestic violence on the Partner Violence Inventory	Differences in methylation between children with CM and those with no CM were observed in methylation sites in the regions of *BDNF, FKBP5, NR3C1*
Yang et al.,^173^	*N* = 192; 50% exposed to CM	5–14 years (*M* = 10.2 years)	58% female	17% European American, 38% Hispanic, 30% African American, 15% biracial	Saliva	CM assessed via child protection records, parent and child reports of trauma using the KSADS interview, child reports on the CTQ, maternal reports of domestic violence on the Partner Violence Inventory	Differential methylation at 2868 CpG sites between children with CM and those with no CM; children with CM had higher methylation at CpG sites with low to mid-range methylation, and lower methylation at sites with high methylation, than children with no CM

*CM* childhood maltreatment, *CTQ* Childhood Trauma Questionnaire, *MCS* Maltreatment Classification System, *ALSPAC* Avon Longitudinal Study of Parents and Children, *KSADS* Kiddie-SADS—Lifetime Version.

**Table 4:** Childhood adversity and studies leveraging epigenome-wide methylation arrays in samples of adults.

Author	Sample	Age(s) at methylation assessment	Gender	Ancestry	Tissue type	Adversity measurement	Adversity-related findings
Han et al.,^87^	*N* = 1130; 72% depressed Characterization of sample: Participants resided in the Netherlands; enrolled in the Netherlands Study of Depression and Anxiety	18–65 years (*M* = 42 years)	65% female	Not reported	Whole blood	Retrospectively assessed trauma via the Netherlands Mental Health Survey and Incidence Study	Childhood trauma was positively associated with epigenetic aging within the depressed group; differences between depressed and control groups for DNA methylation age were replicated in postmortem brain samples
Houtepen et al.,^174^	*N* = 85 for discovery sample (buccal); *N* = 45 for replication sample (blood) Characterization of sample: Enrolled in ALSPAC	18–69 years (*M* = 33 years) in discovery sample; 19–45 years (*M* = 28 years) in replication sample	51% female in discovery sample, 80% female in replication sample	100% Caucasian in discovery sample, 38% Caucasian in replication sample	Buccal cells in discovery sample; Whole blood in replication sample	Retrospectively assessed physical, sexual, emotional abuse, emotional and physical neglect via the CTQ short form; age of onset via the Early Trauma Inventory	Within the discovery (buccal cell) sample, although no sites survived corrections for multiple testing, CM was associated with increased methylation at *KITLG* locus; *KITLG* methylation mediated link between CM and cortisol reactivity; within the replication (blood) sample, CM was associated with increased *KITLG* methylation and cortisol reactivity only in Caucasians; there was no influence of age of onset on methylation
Houtepen et al.,^82^	*N* = 780 in ALSPAC, 66% exposed to adversity; *N* = 552 in NSHD, 68% exposed to adversity	47 years in ALSPAC; 53 years in NSHD	100% female	Not reported	Whole blood in ALSPAC; Buccal cells in NSHD	In NSHD, 5 ACEs prospectively measured via interviews and questionnaires by participants’ mothers, including parental physical illness, parental mental illness, parental death, parental separation, and childhood illness; suboptimal maternal bonding and childhood maltreatment were retrospectively self-reported; in ALSPAC 5 additional ACEs retrospectively assessed via questionnaire: physical, sexual, and emotional abuse, physical and emotional neglect for a total of 11 ACEs	Although no individual CpG sites replicated across cohorts, after correction a total of 97 DMRs were associated with ACE measures in ALSPAC and 134 DMRs were associated with ACE measures in NSHD; even after adjusting for smoking nine differentially methylated regions were associated across both cohorts such that cumulative ACE score associated with methylation variance, as was parental mental illness, parental physical illness, and parental death
Khulan et al.,^175^	*N* = 83 men separated from parents in childhood during wartime and *N* = 83 non-separated controls Characterization of sample: Enrolled in Helsinki Birth Cohort Study	*M* = 64 years for separated group; *M* = 62.9 years for non-separated group	0% female	Born in Finland	Whole blood	Childhood wartime parental separation according to the Finnish National Archives’ register	No association of childhood parental separation and methylation; methylation was associated with the development of depressive symptoms; hypomethylated genes included those with roles in brain development, brain function
Labonté, Suderman, et al., 2012b^176^	*N* = 41; *n* = 25 completed suicide and exposed to severe CM; *n* = 16 controls with no suicide and no CM; *n* = 20 completed suicides with no CM, used for validation Characterization of sample: Quebec Suicide Brain Bank	*M* = 37.3 years in CM/suicide group; *M* = 40.6 years in non-CM/suicide group; *M* = 40.9 years in control group	0% female	100% Caucasians of French-Canadian descent	Brain (hippocampal tissue)	Retrospectively assessed sexual abuse, physical abuse and severe neglect via psychological autopsy, including structured interviews/chart reviews using the CECA adapted for psychological autopsies	CM associated with genome-wide promoter epigenetic alterations; 362 differentially methylated promoters identified in individuals with CM compared with controls--248 showed hypermethylation and 114 showed hypomethylation; observed functional clusters of differentially methylated genes were validated against non-CM suicide completers and were shown to be involved in cellular/neuronal plasticity, with potential candidate marker Alsin (*ALS2*) observed to be differentially methylated
Lawn et al.,^85^	*N* = 989 in ALSPAC, 23% exposed to CM; *N* = 773 in NSHD, 7% exposed to CM	Two timepoints in ALSPAC: 29 and 47 years; 53 years in NSHD	100% female	Not reported	Whole blood in ALSPAC; Buccal cells in NSHD	In NSHD, 5 ACEs prospectively measured via interviews and questionnaires by participants’ mothers, including parental physical illness, parental mental illness, parental death, parental separation, and childhood illness; suboptimal maternal bonding and childhood maltreatment were retrospectively self-reported; in ALSPAC 5 additional ACEs retrospectively assessed via questionnaire: physical, sexual, and emotional abuse, physical and emotional neglect for a total of 11 ACEs	Sexual abuse associated with higher DNA methylation age in ALSPAC at both timepoints (no sexual abuse data available in NSHD); cumulative adversity was not associated with DNA methylation age in ALSPAC or NSHD
Lutz et al.,^84^	*N* = 78 depressed adults who died by suicide; 35% with severe CM	Postmortem	Not reported	Not reported	Anterior cingulate cortex	Severe physical and sexual abuse up to age 15 validated from medical charts, coroner files, and child protective services reports	Hyper- and hypomethylation was detected in the group exposed to abuse compared to the control group; the three most significantly differentially methylated regions intersected with genes directly related to myelin and oligodendrocytes: *LINGO3*, *POU3F1*, and *ITGB1*
Marinova et al.,^177^	*N* = 45; *n* = 30 former indentured child laborers, *n* = 15 controls Characterization of sample: Grew up in rural Switzerland	*M* = 75.9 years in experimental group, *M* = 72.8 years in control group	47% female in experimental group; 53% female in control group	Not reported	Buccal cells	Retrospectively reported former indentured child laborer status	Differential methylation between the two groups, with the strongest difference in *SKAP2*
Marzi et al.,^178^	*N* = 2232 twins; 28% with severe victimization experiences Characterization of sample: Enrolled in Environmental Risk (E-Risk) Longitudinal Study in England and Wales	18 years	Not reported	Not reported	Whole blood	Prospectively assessed exposure to domestic violence; physical, sexual, emotional abuse and emotional and physical neglect; bullying; family violence; cyber victimization; exposure to crime via dossiers that included information from home visit staff, mothers, children, family doctors, and child protection agencies; child completion of Juvenile Victimization Questionnaire	Methylation associated with adversity exposure overlapped with tobacco smoking, thus could not be differentiated
Mehta et al.,^186^	*N* = 169; *n* = 108 with no PTSD (31% with CM), *n* = 61 with PTSD (52% with CM) Characterization of sample: Urban, low-income	*M* = 43.25 years in total sample; *M* = 44.23 years in CM with no PTSD group; *M* = 39.56 years in CM + PTSD group; *M* = 43.69 years in no CM + PTSD group	72% female	89% African American; 11% other	Whole blood	Physical, sexual, emotional abuse, emotional and physical neglect via the CTQ	Gene-expression profiles of PTSD patients with CM were nearly nonoverlapping with CM-exposed controls; these gene expression changes were associated with methylation changes in the same loci in the CM group; functional annotation analyses supported enrichment of central nervous system development and in immune-related tolerance induction pathways in the PTSD group with CM whereas apoptosis and growth rate networks were enriched in the PTSD group without CM
O’Donnell et al.,^83^	*N* = 188; *n* = 99 control group, *n* = 89 Nurse Family Partnership intervention group; 27% exposed to CM; Characterization of sample: Resided in Canada	27 years	53% female in control group; 48% female in NFP group	85% Caucasian in control group; 75% Caucasian in NFP group	Whole blood	Child abuse and neglect retrieved from substantiated reports from child protective services from child ages birth through 15	CM, and participation in NFP, were associated with variation in methylation at age 27; the magnitude of the association between CM and methylation was reduced when accounting for smoking; no significant association between epigenetic age acceleration and CM or participation in NFP
Prados et al.,^179^	*N* = 189; *n* = 96 borderline personality disorder patients with high CM; *n* = 93 depressed patients with low CM Characterization of sample: Participants resided in France and Switzerland	*M* = 36.7 years	78% female	Not reported	Whole blood	Physical, sexual, emotional abuse, emotional and physical neglect via the CTQ	Differential methylation observed in individuals with BPD and high CM compared to individuals with depression and low CM. Patterns of methylation also differed with respect to the severity of CM
Robakis et al.,^180^	*N* = 54 pregnant women recruited from psychiatric clinic	*M* = 32.3 years	100% female	65% Caucasian 11% East Asian 4% South Asian 6% Hispanic 2% African American 11% Multiracial	Buccal cells	Retrospectively assessed physical, sexual, emotional abuse, emotional and physical neglect via the CTQ	CM associated with methylation density in 1580 regions, 162 regions when using FDR < .05; genes included those with metabolic, cellular process, and regulatory functions; CM not associated with mean methylation density over entire captured region of *OXTR*
Roberts et al.,^181^	*N* = 34; 50% with high CM exposure, 15% with medium CM exposure, 35% with no CM exposure Characterization of sample: Enrolled in the Growing Up Today Study	23–29 years (*M* = 26.3 years in no CM group; *M* = 25.4 years in medium CM group, *M* = 25.2 years in high CM group)	100% male	92% white in no CM group, 100% white in medium CM group, 88% white in high CM group	Sperm	Retrospectively assessed physical, sexual, emotional abuse via the CTQ and Conflict Tactic Scales	Differential methylation between those exposed to CM and those that were not; adulthood trauma exposure and mental health partially mediated the association between CM and methylation
Smith et al.,^182^	*N* = 110; *n* = 25 with PTSD without CM, *n* = 25 with PTSD and CM, *n* = 26 with CM, *n* = 34 without CM	*M* = 41.3 years in control group; *M* = 44.1 years in PTSD group	40% female in control group; 37% female in PTSD group	100% African American	Whole blood	Retrospectively assessed sexual, physical, and emotional abuse via the CTQ; stressful life events (e.g., interpersonal stressors, crime, divorce) in the last year or ever via the Stressful Events Questionnaire	No change in global methylation levels in participants with CM or with increased total life stress; methylation was inversely associated with total life stress at one CpG site; no CpG site was associated with CM; in an examination of 60 genes previously identified in the literature, CpG sites in *BDNF* and *CXCL1* were associated with both PTSD and total life stress; differences in methylation profiles as a function of CM did not survive experiment-wide correction for multiple testing
Suderman et al.,^183^	*N* = 40; 30% exposed to CM Characterization of sample: From 1958 British Birth Cohort	45 years	100% male	Not reported	Whole blood and lymphoblast cell lines	Retrospectively assessed verbal, emotional, physical, and sexual abuse via a questionnaire including items derived from the Parental Bonding Instrument, the British National Survey of Health and Development and the US National Comorbidity Survey	Differential methylation associated with CM – both hyper- and hypomethylation; of the differentially methylated genes that perform some regulatory function, most were hypomethylated in CM sample
Tamman et al.,^86^	*N* = 212 veterans	22–93 years (in larger sample of 1,135 veterans)	100% male	100% European American	Saliva	Retrospectively assessed trauma, including child sexual abuse, via the Trauma History Screen	Childhood sexual abuse associated with increased DNA methylation age; greater number of lifetime traumas associated with increased DNA methylation age
Zannas et al.,^184^	*N* = 392 Characterization of sample: Participants enrolled in the Grady Trauma Project	18–77 years (*M* = 41.3 years)	71% female	100% African American	Whole blood	Retrospectively assessed physical, sexual, emotional abuse, emotional and physical neglect via the CTQ	Cumulative lifetime stress (including childhood events) was associated with acceleration of epigenetic aging; childhood abuse exposure alone was not significantly associated with markers of epigenetic aging
Zhang et al.,^185^	*N* = 518, 29% with childhood adversity, 52% with diagnosis of alcohol dependence	Mean age ranged from 33–43 years across groups stratified by alcohol dependence and childhood adversity	56% female	54% African American; 46% European American	Whole blood	Exposure to childhood adversity assessed using four questions from the Semi-structured Assessment for Drug Dependence and Alcoholism	Childhood adversity associated with hypermethylation of seven individual CpG sites (*ALDH1A1, CART, CHRNA5, HTR1B*, *OPRL1, PENK*, and *RGS19)* in European American participants with and without alcohol dependence; associations of childhood adversity and methylation in African American participants were not replicated across those with and without alcohol dependence

*CM* childhood maltreatment, *CTQ* Childhood Trauma Questionnaire, *ALSPAC* Avon Longitudinal Study of Parents and Children, *NSHD* MRC National Survey of Health and Development, *PTSD* post-traumatic stress disorder, *NFP* Nurse Family Partnership, *BPD* borderline personality disorder, *FDR* False Discovery Rate.

### Childhood maltreatment and methylation of candidate genes

#### Children

As displayed in Table 1, 16 empirical articles focused on childhood maltreatment and methylation of candidate genes in children. Most of these studies involved saliva DNA, but one used buccal cell DNA and several examined DNA from blood. The most commonly studied candidate genes were those that regulate glucocorticoid signaling, including *NR3C1* which encodes the glucocorticoid receptor (GR) (six studies represented in eight empirical articles) and *FKBP5* which modulates sensitivity of the GR^24^ (two studies represented in three empirical articles).

Most studies of children support the hypothesis that childhood adversity is associated with higher levels of methylation of *NR3C1*. In our own study of preschoolers, we found that childhood maltreatment status and the number of stressful life events was associated with greater *NR3C1* methylation^25^, and *NR3C1* methylation mediated effects of maltreatment and stressful life events on child internalizing behavior problems^12^. Likewise, in 534 school-aged children with low socioeconomic status, children who experienced maltreatment beginning in infancy or early childhood had greater *NR3C1* methylation than children who had no maltreatment history^26^. Interestingly, there were no differences between children who experienced maltreatment that started in the preschool period or later, and those children with no maltreatment history. However, more chronic maltreatment and more types of maltreatment, were associated with greater *NR3C1* methylation. Using data from the Bucharest Early Intervention Project, time in institutional care was associated with lower levels of methylation of *FKBP5*, which modulates sensitivity of the GR, when children were 12 years old^27^. Lower levels of *FKBP5* methylation in association with childhood maltreatment were also observed in our sample of preschoolers^28^. Another study of Tanzanian children found evidence that maltreatment was associated with hypermethylation of *CRH*, the gene that encodes the hypothalamic corticotropin releasing hormone (CRH) and its downstream target, *POMC*, which encodes the pituitary precursor of ACTH^29^. Taken together, these findings indicate that childhood maltreatment may be associated with altered methylation of genes that regulate the child stress response, suggesting a possible mechanism for the effect of maltreatment and other adversities on poor health outcomes.

Epigenetic alterations with childhood maltreatment have been documented in additional candidate genes. Methylation of serotonin signaling genes, including *SLC6A4*, which encodes the serotonin receptor, and *HTR2A*, the gene that encodes the serotonin receptor subtype, 5-HT_2A_, have been investigated in association with childhood maltreatment and other interpersonal adversities^27,30–32^. Childhood adversity was associated with greater *SLC6A4* methylation in two studies^31,32^, but lower levels of methylation in association with institutional care^27^. Using a subsample (*n* = 785) of the Avon Longitudinal Study of Parents and Children (ALSPAC), Barker et al.^33^ generated an inflammation-related epigenetic polygenic risk score (i-ePGS). Adversity between birth and 7 years of age was related to higher i-ePGS, which, in turn, was an indirect pathway by which adversity was associated with internalizing behavior problems in later childhood. The receptor gene for the pituitary hormone oxytocin (*OXTR*), best known for its role in social behavior and attachment, has also been studied in relation to early adversity in children. Fujisawa et al.^34^ demonstrated greater methylation of *OXTR* in Japanese children and adolescents with a maltreatment history (*n* = 44) compared with controls (*n* = 41), and found that *OXTR* methylation was negatively associated with gray matter volume in the left orbitofrontal cortex.

#### Adults

As displayed in Table 2, 53 empirical articles focused on childhood maltreatment and methylation of candidate genes in adults. Most studies involved analysis of DNA from blood, with a handful of studies examining DNA from saliva or buccal cells, and a few studies of DNA from brain tissue. The most commonly studied candidate genes included *NR3C1* (20 empirical articles), *FKBP5* (eight empirical articles), *SLC6A4* (six empirical articles), and *OXTR* (six empirical articles).

Taken together, the majority of studies focused on *NR3C1* methylation observed significant associations of childhood maltreatment and methylation of this gene, but there is considerable variability in the findings. Several studies found increased *NR3C1* methylation with childhood maltreatment, but others found no effect, or reduced methylation with maltreatment. Melas and colleagues^35^ found that childhood adversity was associated with saliva DNA *NR3C1* hypermethylation, with childhood parental loss linked to higher methylation near a transcription factor binding site. Two reports analyzed DNA from the Quebec Suicide Brain Bank in relation to childhood maltreatment retrospectively assessed via psychological autopsy, including a group with suicide and childhood maltreatment, a group with suicide but no maltreatment, and a control group. The suicide with maltreatment group had greater hippocampal *NR3C1* methylation and reduced *NR3C1* expression across exon 1_F_^36^, reduced *NR3C1* gene expression across exons 1_B_, 1_C_, and 1_H_, and reduced *NR3C1* methylation at exon 1_H_^37^, in comparison with the other two groups. In contrast, both suicide groups differed from controls in regions of exon 1_B_, with hypermethylation at two CpG sites and hypomethylation at one CpG site^37^. In a diverse sample of adults, childhood maltreatment was associated with greater methylation of leukocyte *NR3C1*, and maltreatment was associated with lower *NR3C1* gene expression^38^. Likewise, in two samples with psychiatric disorders, childhood maltreatment was associated with greater *NR3C1* methylation^39,40^. In contrast, in a recent study, we found that early adversity, and the number of adverse exposures, was associated with lower *NR3C1* methylation in healthy adults^41^. Other studies found no association of maltreatment and *NR3C1* methylation^42–46^, but one of these found that *NR3C1* methylation moderated effects of maltreatment on cortisol response to the Trier Social Stress Test.

The literature focused on *FKPB5* methylation in adults also demonstrates inconsistent effects. Klengel et al.^47^ found that childhood maltreatment was associated with lower methylation of *FKBP5* in adults with the rs1360780 T risk allele, and this effect was observed in both a subsample of participants from the Grady Trauma Project and a replication sample. This effect was more recently replicated by Tozzi and colleagues^48^ who also reported that childhood maltreatment was associated with lower *FKBP5* methylation among those with the T risk allele of the rs1360780 SNP. In contrast, Harms et al.^49^ found significant *positive* associations between stress in childhood and methylation of *FKPB5* in young adulthood using a prospective longitudinal design, and a number of studies did not find significant associations among childhood maltreatment and *FKBP5* methylation^50–53^, or moderation by FKBP5 risk allele^50,51^.

Turning to the serotonin system, several studies have examined whether childhood maltreatment is associated with methylation of *SLC6A4*, the gene for the serotonin transporter. Most studies have found a positive association of childhood maltreatment with methylation of regions of this gene^54–58^ although some studies show equivocal or trend-level effects^59,60^ or no effect^61^ of maltreatment on *SLC6A4* methylation.

Based on the hypothesis that oxytocin may play a protective role in the biological response to stress and trauma^62^, five studies investigated methylation of the oxytocin-receptor-gene (*OXTR)* in adulthood; all five reported no overall association of childhood maltreatment with *OXTR* methylation. A number of these studies did report indirect or moderation effects. In a sample of 309 African American men, childhood adversity had a significant indirect effect on *OXTR* methylation through socioeconomic instability^38^. No significant direct effect of childhood adversity on *OXTR* methylation was observed. Likewise, while Smearman and colleagues^63^ did not observe simple associations of child abuse history and *OXTR* methylation when accounting for multiple comparisons in their sample of 393 African American adults, *OXTR* methylation moderated the association of childhood abuse and psychiatric symptoms.

Several other candidate genes have been examined in individual studies. For example, childhood trauma was associated with lower methylation of the proinflammatory *IL-6* promoter, and lower methylation in turn was associated with greater salivary IL-6 in response to the Trier Social Stress Test^64^. Although this study drew upon a relatively small sample, the findings are consistent with other work demonstrating effects of childhood trauma and maltreatment on inflammatory processes.

### Childhood maltreatment and epigenome-wide association studies

Arising in part out of replication inconsistencies as well as interest in identifying novel variation, the past decade has included an increase in the number of Genome Wide Association Studies (GWAS), which are agnostic by design and interrogate genetic variation across millions of common genetic variants the entire genome. However, a limitation of GWAS is the extremely large samples needed to detect effects after adjusting for multiple testing. Similar to GWAS approaches, epigenome-wide association studies interrogate markers across the entire genome. Consideration of the biology of DNA methylation as well as optimization of power has led to the development of new frameworks for analyzing multiple markers, including examination of CpG sites that are in close proximity to one another (differentially methylated regions, DMRs), examination of DNA methylation in biological pathways known to be involved in the condition, and application of analytic tools that use a ranking approach rather than relying on p values. Researchers have also developed quality control standards and approaches for addressing both concerns with Type I error as well as population stratification that may arise in diverse samples^65^.

Given that biological processes function as part of a larger interrelated system, research has increasingly focused on how either systems or patterns of alterations in methylation may be important in efforts to understand epigenetic modifications. Studies have begun to explore DNA methylation as a measure of molecular aging, with evidence that this epigenetic “clock” is associated with age-related disorders and mortality^66–70^ as well as with age-related development such as menopause^71,72^. Several analytical methods have been developed to measure epigenetic age as indicated by epigenome-wide methylation profiles^73–77^. This emerging area of research has the potential to provide exciting evidence for understanding the impact of early adversity on molecular age.

#### Children

As displayed in Table 3, 12 empirical articles focused on epigenome-wide effects of maltreatment and other adversities in children. These studies suggest that maltreatment is associated with variation in methylation across the genome, and several differentially methylated regions and genes have been identified. In a racially and ethnically diverse sample of 548 children, Cicchetti et al.^78^ found differential whole-genome methylation in children with a maltreatment history relative to children with no maltreatment. Maltreated children had higher levels of methylation at sites where methylation was generally low, and lower methylation at sites where methylation was generally high, compared to non-maltreated children. Using data from a subsample (*n* = 774) of the ALSPAC, Dunn et al.^79^ examined associations among childhood adversity and epigenome-wide methylation in children at 7 years of age. Thirty-eight differentially methylated CpG sites were identified in association with early adversity, and the developmental timing of adversity was the most salient predictor of methylation. Neither the simple presen-ce/absence of adversity, recency of adversity, nor accumulation of adverse experiences was associated with altered methylation.

Studies of early adversity and epigenetic age are just emerging in samples of children. Very recently in a sample of 247 children who were 8–16 years of age, threat-related early adversity (including abuse and violence exposure), but not deprivation (including neglect), was associated with accelerated DNA methylation age^80^. Furthermore, threat-related early adversity exerted a significant indirect effect on depressive symptoms through accelerated DNA methylation age, suggesting that this measure of early adversity is clinically relevant to psychiatric outcomes. This is consistent with Jovanovic et al.^81^ that demonstrated that violence exposure was associated with greater DNA methylation age acceleration in African American children who were 6–13 years of age.

#### Adults

As displayed in Table 4, 19 empirical articles focused on epigenome-wide effects of childhood maltreatment in adults. Collectively, these studies suggest that childhood maltreatment exerts epigenetic effects across the genome, yet these studies differed in their overall focus and methodology. In the ALSPAC and the MRC National Survey of Health and Development, Houtepen et al.^82^ identified nine differentially methylated regions (DMRs) in the genome that replicated across the two cohorts and were associated with childhood adversity. No individual CpG sites in their epigenome-wide analysis replicated across the cohorts. O’Donnell et al.^83^ used a different methodological approach to describe variation in DNA methylation, and found that childhood maltreatment was associated with variation in methylation at 27 years of age utilizing principal components scores to describe variation in methylation. Lutz et al.^84^ examined genome-wide DNA methylation and gene expression in postmortem brain samples of adults with a history of depression who died by suicide. This study found that child abuse history was associated with differential methylation specifically in oligodendrocytes in the cingulate cortex, as well as expression of myelin-related genes.

Investigations using epigenome-wide data to explore effects of early adversity on accelerated epigenetic aging have shown mixed outcomes. Lawn et al.^85^ used the ALSPAC and the MRC National Survey of Health and Development to examine associations of childhood psychosocial adversity, including abuse and neglect, and DNA methylation age acceleration. Childhood sexual abuse was associated with methylation age acceleration in ALSPAC, and this effect remained significant when controlling for socioeconomic position. Data regarding sexual abuse was not available in the MRC survey, however Tamman et al.^86^ also found that childhood sexual abuse was associated with increased DNA methylation age. Neither individual adversity types nor a cumulative measure of adversity were associated with methylation age acceleration in ALSPAC and the MRC National Survey of Health and Development^85^. Han et al.^87^ also reported a positive association between a history of childhood trauma and epigenetic aging in adults with MDD. DNA methylation age was also examined in the sample of 27 year olds described above, with analyses revealing no differences in epigenetic age as a function of childhood adversity^83^. Although there have been more studies focused on childhood adversity and accelerated epigenetic age in adults, few studies overall have been completed.

## Discussion

**Fig. 2:**
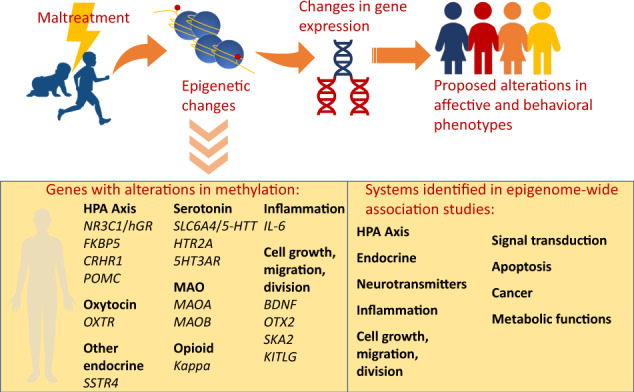
Conceptual diagram representing the epigenetic pathway from maltreatment to health and mental health outcomes. Note: This review presents data focused on the relationship between maltreatment and alterations in methylation, whereas the proposed alterations in affective and behavioral phenotypes are explored further elsewhere.

This systematic review examined associations of childhood maltreatment and DNA methylation in children and adults. One hundred empirical articles focused on humans were identified. These studies included both candidate gene and epigenome-wide approaches. Strengths of the literature included: (1) rigorous approaches to measure childhood maltreatment in studies focused on DNA methylation in children, including record review methods; (2) several racially and ethnically diverse samples in the child studies; (3) diverse and innovative approaches to measuring epigenome-wide effects of maltreatment, including exploration of how early adversity may lead to epigenetic age acceleration; and (4) several replication studies focused on childhood maltreatment and methylation of glucocorticoid signaling genes in children and adults. Collectively, these studies provide evidence that childhood maltreatment and other adversities are associated with DNA methylation. Genes and pathways observed to have altered methylation in relation to childhood maltreatment, and the expected epigenetic pathways from maltreatment to health and mental health outcomes, are displayed in Fig. 2.

Studies of childhood adversity and DNA methylation often focused on methylation of candidate genes that regulate glucocorticoid signaling including *NR3C1* and *FKBP5*. Most studies in children demonstrated increased *NR3C1* methylation with maltreatment. In adults, several studies documented greater *NR3C1* methylation in those exposed to childhood maltreatment, whereas others demonstrated no associations of maltreatment and *NR3C1* methylation. Furthermore, in our own recent work we found lower *NR3C1* methylation in association with childhood adversity^41^, and other studies have identified CpG sites in *NR3C1* that are hypomethylated in association with maltreatment or suicide^37^ and post-traumatic stress disorder (PTSD)^88,89^. For *FKBP5*, Klengel and colleagues first published findings of an interaction of childhood maltreatment and *FKBP5* genotype such that maltreatment was associated with lower *FKBP5* methylation in adults with the T risk allele^47^. Lower *FKBP5* methylation with maltreatment—although not interacting with genotype—has been seen in the two studies with published data in children. The maltreatment by genotype interaction predicting lower *FKBP5* methylation in adults was recently replicated^48^, but other studies have not observed an effect of maltreatment, or an interaction of maltreatment and genotype, and one study of young adults found that stressful life events in childhood were associated with greater *FKBP5* methylation, which in turn mediated effects of early stress on prefrontal brain activity^49^.

Many studies in adults utilized samples with chronic disease and mental health diagnoses, and did not exclude participants with consistent medication use, which may have influenced the findings. Overall levels of methylation of the *NR3C1* gene promoter are generally very low, and this may also limit the ability to reliably detect group differences. However, there is evidence that *NR3C1* methylation at exons 1_F_, 1_B_, and 1_C_, is inversely associated with GR gene expression^36,37^. Since GR mediates negative feedback to the HPA axis, higher methylation—and lower GR gene expression—is expected in conditions with elevated cortisol and there is some evidence for this^90,91^. But variable findings for methylation of *NR3C1* and *FKBP5* are perhaps not surprising given that maltreatment, trauma, and stress-related psychiatric disorders have been linked to both exaggerated and diminished basal and provoked cortisol concentrations, which may in part depend on measured or unmeasured individual and trauma characteristics^92,93^. Moreover, many genes and gene products regulate HPA axis function, and complex interactions among participant characteristics (including age and genetic ancestry), genotype (with allele frequencies that may differ based on genetic ancestry), and stress exposure may best characterize these effects.

Interaction effects may also characterize methylation of oxytocin system genes, given findings of indirect or moderation effects in the absence of simple effects^63,94^. Although the potential risk for statistical artifacts provide important cautions to investigations exploring interaction effects, interactions and conditional effects are known to occur in biological systems and may be appropriate to examine in large samples and/or using stratification or model invariance strategies. Methylation of serotonin signaling genes was also examined in multiple studies. Most studies of adults and some work in children found elevated methylation of *SLC6A4* with maltreatment, but there are also reports of lower *SLC6A4* methylation at some CpG sites with adversity, or no difference between adversity groups. Differences in epigenetic findings in *SLC6A4* as well as other candidate genes may also be related to inconsistencies in the ways that the number of differentially methylated regions are assessed. For example, *SLC6A4* has 81 CpG sites with studies adopting a range of approaches to assessing this large region, including selection of target sites based on the literature, multiple testing of sites, and strategies for binning methylation at multiple sites^95^. Given evidence for ancestry-related differences in methylation, differences across studies related to ancestry may also potentially contribute to apparent inconsistencies^96^.

Studies using agnostic approaches to exploring multiple markers across the epigenome also suggested that maltreatment is associated with variation in methylation, though these epigenome-wide studies have generally failed to identify commonly studied candidate genes, leading to questions about candidate approaches. Several reasons for these inconsistencies have been advanced, including large effect sizes needed to detect an effect after multiple testing corrections, biological considerations such as the potential combinatory impact of genotype and DNA methylation (see ref. ^83^ for an example of integration of genotype and epigenetic influences), interactions among systems of genes, and differences in aspects of the phenotyping and heterogeneity of different studies. Due to traditional approaches to corrections for multiple testing, studies that analyze methylation of multiple markers across the genome tend to require large samples which are often heterogeneous and may have less intensive phenotyping and measurement of maltreatment and other relevant exposures. Promising markers identified with epigenome-wide approaches show some consistency in terms of their roles in neural cell development (*BDNF, KITLG*, and *POU3F1)*, signaling and apoptosis (*LINGO3* and *2NPFF2)*, neural influences on movement (*ALS2*), neuroinflammation (*ITGB1)*, and some tentative evidence for immune markers (*CXCL1)*.

A recent area of epigenome-wide research involves DNA methylation as an indicator of molecular age. A number of analytic strategies have been used to examine how early life trauma (and/or symptoms associated with trauma such as depression or post-traumatic stress disorder) may accelerate aging. A number of adult and child studies find age acceleration among individuals exposed to maltreatment (meaning that DNA methylation age is older than the chronological age). Childhood maltreatment and other adverse exposures alter epigenome-wide profiles, thereby likely contributing to chronic disease and physical aging^97–101^. Interestingly, the particular markers observed to be relevant in methylation age acceleration research may point toward biological systems implicated in the link between maltreatment and disease. For example, the method described by Horvath includes CpG sites enriched for glucocorticoid response elements^99^. New methods for capturing DNA methylation age continue to be developed, and very recently Belsky and colleagues^102^ reported a new algorithm that was derived from longitudinal data of 18 biomarkers of organ-system integrity to capture the rate of aging up to the time of measurement. This new algorithm, but not other previously established measures of DNA methylation age, was associated with childhood maltreatment in sample of 1658 young adults who were longitudinally followed since childhood. As new methods to capture age acceleration continue to develop, systematic reviews and meta-analytic approaches will continue to be important to synthesize associations of child maltreatment and the range of measures of methylation age.

Studies of maltreatment and methylation in children were often characterized by careful measurement of adverse exposures using structured record review techniques and in-depth interview methods, and they often utilized prospective and longitudinal designs. In children, DNA methylation was most frequently measured in saliva or buccal epithelial cells, with fewer studies measuring methylation in blood. Many of the samples were racially and ethnically diverse, and these studies covered the full developmental spectrum, from early childhood through adolescence. In contrast, nearly all the adult studies drew upon retrospective reports of childhood maltreatment. Utilization of prospective longitudinal designs and record review techniques would address a significant gap in the literature. Furthermore, in contrast to studies of maltreatment and methylation of candidate genes in children, most adult studies measured methylation in leukocyte DNA.

Moving beyond simple effects of maltreatment on DNA methylation, several studies highlighted the importance of the developmental timing of exposure to adversity^26,27,78,79^. More research is needed to understand how early adversity at each developmental epoch may be associated with differences in epigenetic marks. Moreover, longitudinal research is needed to explore whether epigenetic changes secondary to early adversity may “reset” during later developmental periods or perhaps may be impacted by later experiences, which either attenuate or compound early adversity. Retrospective research with adults who report childhood adversity has numerous benefits in terms of cost- and time-effectiveness; however, reliance on retrospective reports of childhood adversity present important methodological challenges related to recalling the timing of childhood events, ability to recall very early life events, and systematic recall biases, introducing measurement limitations that are difficult to overcome^103^. Many studies use the Childhood Trauma Questionnaire^104^ or other self-report measures. Some self-report measures, such as the Traumatic Life Events Questionnaire^105^ and the Maltreatment and Abuse Chronology of Exposure^106^, include trauma characteristics such as the age or frequency of occurrence, which may be important determinants of epigenetic and phenotypic outcomes. Future research may also benefit from interview approaches that can help with retrospective recall such as participant-tailored anchoring.

Although many adult studies reviewed here relied on recall, much can be learned from the few studies that capitalized on longitudinal cohorts. For example, Harms et al.^49^ assessed stress when children were 9–13 years old and then methylation approximately 10 years later. O’Donnell et al.^83^ assessed childhood maltreatment in the first 15 years of life and methylation at age 27. Future research should capitalize on existing studies of children that utilized rigorous measures of the environment as they develop over time to expand the literature in adults. Likewise, very few studies drew upon repeated assessments of DNA methylation over time. In our own research, we demonstrated that childhood maltreatment and other adversities are associated with change in methylation of glucocorticoid signaling genes over time in early childhood^13,107^. Future research should further examine maltreatment as a predictor of change in methylation across development.

Importantly, the majority of studies focused on exposure to childhood maltreatment without consideration of the prenatal environment. Prenatal exposures exert epigenetic effects on several biological systems, particularly the development of the child stress response. For example, intimate partner violence in pregnancy is associated with increased *NR3C1* in late childhood and adolescence^108^. Smoking and depression in pregnancy have also been associated with altered methylation of placental stress-related genes, such as *NR3C1* and *HSD11B2*, which encodes the enzyme that inactivates cortisol^109–112^. Associations of childhood maltreatment and DNA methylation may be partially accounted for by prenatal environmental factors. Conversely, maltreatment may exert a unique and independent effect on DNA methylation above and beyond prenatal exposures. Barker et al.^33^ found that child adversity between birth and 7 years of age was associated with an inflammation-related epigenetic polygenic risk score (i-ePGS) at age 7, but there was no association of prenatal adversity and the i-ePGS. Future research should disentangle prenatal exposures and adversities experienced in childhood to better understand effects of maltreatment on DNA methylation in both childhood and adulthood.

Very few studies examined sex differences in the effects of childhood maltreatment on DNA methylation. Studies focused on in utero stress exposure often find sex differences in epigenetic pathways and observed outcomes in both preclinical and human models^113–115^. For example, Braithwaite et al.^116^ found that maternal depressive symptoms in pregnancy were associated with greater methylation of *NR3C1* in male, but not female, infants. Stroud et al.^109^ found that the moderating effects of placental *HSD11B2* methylation on links between prenatal major depressive disorder and infant cortisol response emerged most strongly for newborn daughters, whereas direct and moderating effects of *SLC6A4* gene expression were evident only for sons^109^. In one of the few studies identified in this review that examined sex differences, Cicchetti et al.^78^ found that boys and girls showed different directions of the effect of maltreatment on methylation of *ALDH2*, a gene that encodes a key enzyme in the metabolism of alcohol. Sex differences were also observed in the effect of the developmental timing of adversity. As we have shown in a meta-analysis with *ADCYAP1R1*, the adenylate cyclase activating receptor gene associated with PTSD, depending on the functional outcomes of the gene(s), sex and developmental differences are reasonable to expect^117^. Future work on maltreatment and DNA methylation should consider the role of child sex to ensure that important moderation effects are not being overlooked.

An additional future direction is to move from association to causal inference. Maltreatment is often confounded with additional measures of adversity, including poverty and other sociodemographic factors, as well as personality factors, and potentially genetic factors. Many studies reviewed utilized statistical control for potential confounding factors; however, true random assignment designs are not ethical in humans. Thus, synthesizing across preclinical studies (where random assignment is possible) and human association studies will be critical. Additionally, innovative designs in human studies, including random assignment to interventions to reduce maltreatment, and control groups that are demographically and psychosocially matched to maltreatment groups, may allow the field to move closer to causal inference. Research considering intervention effects on change in methylation over time may also address this gap in knowledge. Indeed, we found that service utilization was associated with increases in *FKBP5* methylation over time in preschoolers with early adversity^107^. More recently, Vinkers et al.^118^ observed changes in methylation among soldiers successfully treated for PTSD such that changes in methylation were observed among soldiers who had reductions in PTSD symptoms. Taken together, this work provides initial evidence that psychosocial interventions exert influence on the epigenome.

DNA methylation has been measured in several tissue types, including blood, saliva, and buccal cells. Yet, the majority of studies focused on DNA methylation in children utilized saliva and buccal cells, and fewer examined methylation in blood. Although some researchers have questioned the value of peripheral markers in research aimed at understanding psychobehavioral outcomes believed to be related to central brain processes, with some researchers suggesting that psychiatric epigenetic research should be limited to brain tissue, a number of studies point to the value of peripheral indicators. Research has shown reasonable concordance between gene-expression signals in blood and brain^119–121^ and primate research has identified correspondence in DNA methylation profiles in the brain and blood^122^. Similar gene expression was found in the cerebellum and peripheral blood mononuclear cells (PBMCs) across 4000 unique genes^123^. Correspondence between DNA methylation in PBMCs and postmortem brain tissue was identified with respect to a marker of reward and stress-induced responses^124^. Recent research showed good correspondence of DNA methylation in blood and saliva^125^. Studies of methylation age have also shown consistency across tissue types^75^. Interestingly, there is some evidence^126^ that saliva samples may be more strongly associated with brain methylation than blood samples, although correlations of methylation in the brain with saliva, blood, and buccal cell DNA were all observed to be high and the strength of these associations appear to depend on the genomic region of interest^127^. These studies have provided helpful signals regarding the utility of blood, brain, saliva, and buccal cells, and Epigenomic Roadmap datasets now provide some insights into the ways that peripheral methylation profiles may map on to those in brain tissue^128,129^. Nonetheless, cell type heterogeneity remains a significant challenge for epigenetic research that has been addressed using a number of strategies such as cell counts, cell separation and examination of single cells, and analytic strategies that account for cell type^130–133^.

Another concern involves the technology and analytic strategies used to interrogate genome methylation. Power to detect true differences and false positives are both major concerns, so large samples sizes and replication samples are required, but very large studies may not be able to provide rich data on maltreatment. Researchers have also described the importance of considering reliability of BeadChip technology, the most frequently applied technology for interrogating the genome, as a function of: (a) sample type, with lower reliability and replicability in dried blood spots, (b) tissue type, with some probes demonstrating greater cross-tissue concordance, (c) platform (such as Ilumina Infinium HumanMethylation450 BeadChip with nearly 500,000 sites vs Ilumina MethylationEPIC BeadChip with nearly 850,000 sites), and (d) observed variability at each site, with lower reliability at sites with less variability^134–137^. Research is needed to carefully document reliability considerations for proper assessment of reproducibility; interested readers are directed to a thorough discussion of reliability, replication, and reproducibility in DNA methylation measurement (see ref. ^134^). In addition to the importance of clear site level documentation of findings in publications, suggestions for addressing reliability concerns in ongoing research include analytic approaches such as dropping CpG sites with low observed variability, replicating findings with procedures such as pyrosequencing, and integrating preanalysis reliability metrics^134,136,137^. Focusing the analyses on regions with more variability across participants reduces the likelihood of false positives, and also reduces the number of comparisons, thereby increasing power. It is also important to note that methylation arrays assess only CpG sites; this yields fewer sites than whole-genome bisulfite sequencing approaches, and some research suggests some important variability occurs outside of these CpG islands^137,138^. Standards in this field continue to be refined, and researchers should follow these developments closely. At a minimum, researchers should implement strategies to address quality control (including thorough evaluations of normalization procedures and strategies for addressing batch effects), cell heterogeneity, and sample ancestry^65,139–141^.

**Fig. 3:**
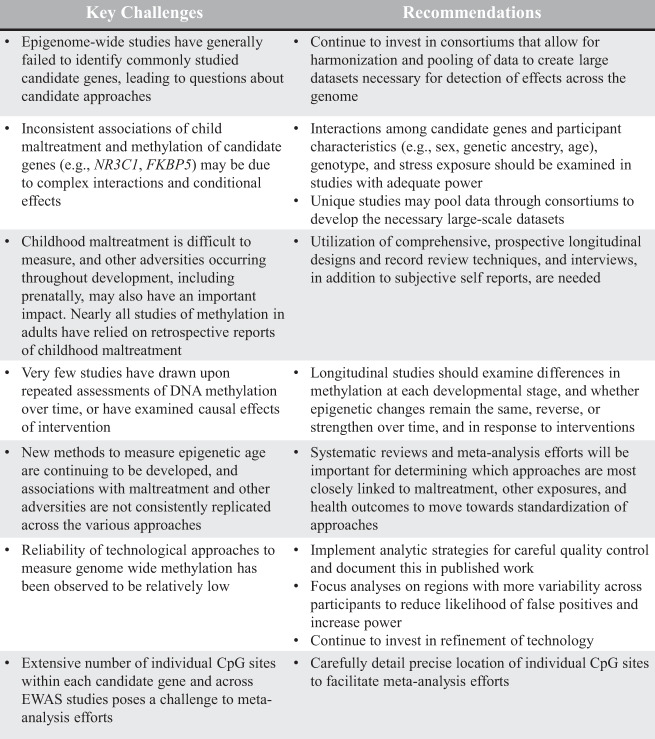
Key challenges and recommendations. Note: Key challenges facing the field and associated recommendations for future research.

Our systematic review examined associations of maltreatment and other interpersonal adversities during childhood with DNA methylation in children and adults. We identified 100 studies, including studies utilizing both candidate gene approaches and epigenome-wide analyses. Several strengths of the literature were identified, as well as directions for future research. Key challenges facing the field and associated recommendations are described in Fig. 3. We included both studies focused on childhood maltreatment and other adversities, as well as studies that were not focused specifically on early adversity but included measurement of adversity in their examination of another topic (e.g., psychiatric or physical health diagnosis). Inclusion of these studies represents both a strength and limitation due to selection biases inherent to these designs, as well as confounds such as the presence of medical conditions and medications that may influence methylation. We acknowledge several other limitations to the current review. Specifically, (1) the present review focused on DNA methylation and did not include studies addressing other epigenetic modifications (e.g., microRNA, histone modifications); (2) the review did not address the functional impact (e.g., gene expression, proteins) of DNA methylation alterations in various studies; and (3) although systematic, the review was qualitative, and thus, does not provide information regarding effect sizes or heterogeneity of included studies. Meta-analytic reports of the most commonly studied candidate genes and EWAS results would build upon our qualitative analysis; however, the extensive number of individual CpG sites both within each candidate gene and across the EWAS studies poses a significant challenge. Furthermore, a critical limitation of the literature is that the location of individual CpG sites is often not consistently identified in empirical manuscripts, further precluding meta-analytic approaches. Future research should more carefully detail the precise location of CpG sites to facilitate future meta-analysis efforts.

In less than two decades, there has been an explosion of research in childhood maltreatment and epigenetics. Mirroring trends observed in genetic research, studies have increasingly focused on sampling methylation across the genome. As with genetic research, advantages of this approach include opportunities to identify key mechanisms that would otherwise have been overlooked. However, this approach generally requires larger samples to account for multiple testing which in turn has been criticized for less nuanced phenotyping of samples permitted in smaller cohorts. Some of these concerns may be somewhat addressed through collaborations such as efforts undertaken by the Psychiatric Genetics Consortium working groups, particularly when care is taken to balance genomic inflation/deflation and to address concerns that can arise with diverse samples interrogated on differing platforms^65^. Researchers have also begun to explore ways that methodological approaches may improve understanding of epigenetic factors and adversity outcomes such as repeated measurement, sampling across tissues, twin/family approaches, and psychological/pharmacological treatments. Epigenetic approaches are also perhaps best understood in combination with thorough assessments of genotype, epigenome, and gene expression. Many factors related to the type and timing of adversity, availability and quality of buffers to adversity, and new events and time since early adversity are likely critically important influences on negative outcomes of adversity. Ongoing recognition of the complexity of biological and environmental factors as well as phenotypic nuances is critically needed—apparent inconsistences do not necessarily negate findings from either of the divergent studies but rather may point to important considerations for ongoing research and theoretical conceptualizations.
